# Advancements and Challenges in Antenna Design and Rectifying Circuits for Radio Frequency Energy Harvesting

**DOI:** 10.3390/s24216804

**Published:** 2024-10-23

**Authors:** Martins Odiamenhi, Haleh Jahanbakhsh Basherlou, Chan Hwang See, Naser Ojaroudi Parchin, Keng Goh, Hongnian Yu

**Affiliations:** School of Computing, Engineering and the Built Environment, Edinburgh Napier University, Edinburgh EH10 5DT, UK; haleh.jahanbakhshbasherlou@napier.ac.uk (H.J.B.); c.see@napier.ac.uk (C.H.S.); k.goh@napier.ac.uk (K.G.); h.yu@napier.ac.uk (H.Y.)

**Keywords:** RF energy harvesting, antenna design, rectifying circuit, nonlinearity, IoT applications, wireless power transfer

## Abstract

The proliferation of smart devices increases the demand for energy-efficient, battery-free technologies essential for sustaining IoT devices in Industry 4.0 and 5G networks, which require zero maintenance and sustainable operation. Integrating radio frequency (RF) energy harvesting with IoT and 5G technologies enables real-time data acquisition, reduces maintenance costs, and enhances productivity, supporting a carbon-free future. This survey reviews the challenges and advancements in RF energy harvesting, focusing on far-field wireless power transfer and powering low-energy devices. It examines miniaturization, circular polarization, fabrication challenges, and efficiency using the metamaterial-inspired antenna, concentrating on improving diode nonlinearity design. This study analyzes key components such as rectifiers, impedance matching networks, and antennas, and evaluates their applications in biomedical and IoT devices. The review concludes with future directions to increase bandwidth, improve power conversion efficiency, and optimize RF energy harvesting system designs.

## 1. Introduction

Electronic devices and wireless sensors play critical roles in healthcare, sports monitoring, environmental sensing, wearable technology, and implants. However, their widespread adoption is limited by the constraints of conventional batteries, which have short lifespans and are difficult to replace in many use cases [1,2]. Addressing these issues is crucial for the longevity of Internet of Things (IoT) devices, which require reliable and sustainable energy supply strategies [3,4]. Although techniques such as data aggregation and sleep scheduling have enhanced operational efficiency, there remains an increasing demand for wireless energy harvesting (WEH) solutions that can harness far-field radio frequency (RF) energy [4,5,6,7]. Several specific applications in IoT can benefit from RF energy harvesting. In smart homes, RF energy can power wireless sensors and devices, enabling continuous communication and monitoring without the need for frequent battery replacements. Similarly, wearable health monitoring systems can leverage ambient RF energy to operate autonomously for extended periods, reducing maintenance and improving patient care. Additionally, RF energy harvesting is pivotal in smart agriculture, where IoT-based sensors monitor environmental conditions across large, remote areas. Finally, logistics and supply chain systems that rely on RFID and IoT-based tracking networks benefit from the extended lifespan provided by RF energy harvesting.

Recently, research has been conducted in the field of RF energy harvesting. However, the majority of surveys predominantly center on specific aspects of rectenna circuit design. To comprehensively comprehend recent advancements in RF technology, including antenna arrays, artificial intelligence-assisted antenna design, and metamaterials, one needs to broaden the scope of reviews. Surveys [8,9] have investigated present patterns and forthcoming paths for RF energy-gathering gadgets and circuits. These assessments underscore methods and approaches to improve the RF-to-DC conversion efficiency of rectenna systems, with a specific concentration on antenna arrays, impedance networks, and rectifiers. However, the incorporation of metamaterials and artificial intelligence in rectenna design remains largely unexplored in these surveys and approaches to enhance the RF-to-DC conversion efficiency of rectenna systems, with a specific concentration on antenna arrays, impedance matching networks, and rectifiers. However, the incorporation of metamaterials and artificial intelligence in rectenna design remains widely unexplored in these surveys. Contrarily, a review by Mohammad et al. [10] and Bashar et al. [11] thoroughly examined current developments in metamaterial applications in antenna design. It studied planar antenna loading and composite right/left transmission in the literature. The focus is not on impedance matching and rectifier design but rather is restricted to antenna design. Musa et al. [12] reviewed the design of reconfigurable antennas using metamaterials. However, their work did not explore essential topics such as artificial intelligence, rectifiers, and the crucial synergy between the antenna, impedance matching network, and rectifiers. These areas require further exploration to improve the design of reconfigurable antennas. A previous review [13] focused on employing CMOS RF-DC rectifiers for a wide power dynamic range, but it did not cover the broader concept of RF energy scavenging. Despite the abundance of literature on RF energy harvesting and its technology, only a few surveys have comprehensively reviewed all the key recent technologies, such as metamaterial-inspired antenna designs, Schottky diode, rectenna, and power generated in RFEH design. While these reviews provide valuable insights, they tend to focus on specific components, overlooking the broader integration of related advancements such as artificial intelligence (AI), antenna arrays, and Schottky diode RF-DC rectifiers. Moreover, previous reviews have not comprehensively addressed the challenges of efficiency, miniaturization, and fabrication in ambient energy harvesting systems.

This review aims to fill these gaps by providing a comprehensive examination of radio frequency energy harvesting (RFEH) systems, including their structures, features, and applications. It discusses key challenges in rectenna design, bandwidth enhancement, and miniaturization, while also exploring the role of AI-assisted antenna design and metamaterials in enhancing performance. Furthermore, the review considers the integration of CMOS and Schottky diode rectifiers, offering a holistic perspective on RFEH for future electronic devices and sensors.

The rest of this paper is organized as follows: Section 2 covers energy harvesting techniques, focusing on the history and evolution of wireless power transfer (WPT) and RF transmission. Section 3 examines critical parameters in antenna technology, with an emphasis on improving bandwidth, gain, and radiation patterns. Section 4 discusses impedance matching networks and their associated challenges. Section 5 reviews advancements in CMOS and Schottky diode rectifiers, Section 6 focuses on power consumption of sensors and energy harvested from rectenna, and Section 7 concludes with future prospects and challenges for RFEH technology.

## 2. Energy Harvesting System

**Figure 1:**
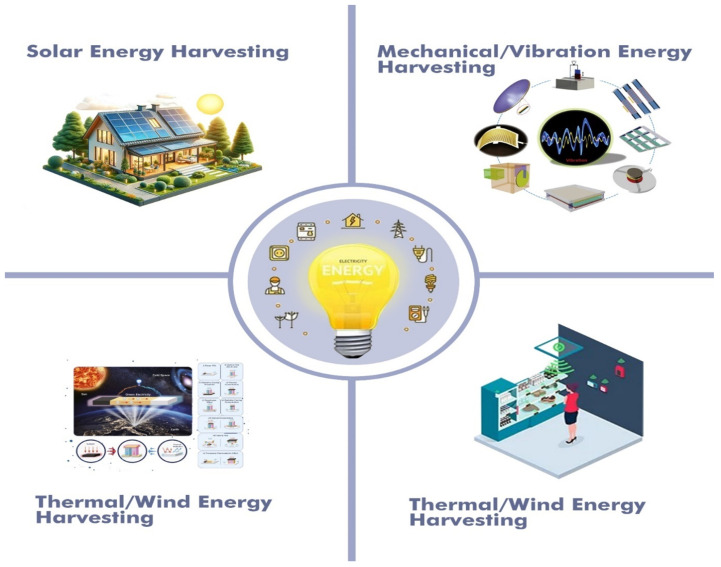
Various energy harvesting techniques.

Ambient energy harvesting extracts power from surrounding sources, providing a critical energy solution for portable electronics and autonomous sensors [14]. Various energy sources each offer unique advantages and limitations. Figure 1 presents an overview of several energy harvesting methods, including solar, thermal, mechanical, and RF-based approaches, highlighting their respective operating principles and applications.

Solar energy converts sunlight into electricity via photovoltaic cells, making it a sustainable option for outdoor applications. However, its efficiency depends on sunlight intensity and duration, with typical conversion rates around 8% [15]. Thermal energy harvesters exploit temperature differences using the thermoelectric effect. These systems power devices such as health monitors and smartwatches, though they face lower conversion efficiencies [16].

Wind energy harvesters convert wind into electricity using turbines, providing a clean and renewable power source. However, the intermittency of wind flow poses significant challenges to its efficiency [17].

Mechanical energy harvesters capture energy from motion and vibrations through electrostatic, piezoelectric, and electromagnetic mechanisms. These systems harvest energy from activities like walking or from industrial vibrations [18].

RF energy harvesting has gained prominence due to the rise of wireless communication technologies, including IoT and 5G networks. Near-field RF harvesters use specialised sources to achieve high energy transfer efficiencies, often exceeding 80%. Far-field RF harvesters capture signals from distant sources such as cellular towers, converting them into usable power through rectifier circuits. The power density and efficiency of this method depend heavily on the signal propagation distance and source output power [19].

Table 1 compares these energy harvesting methods, evaluating their availability, power density, characteristics, efficiency, applications, and associated benefits and limitations. This comprehensive comparison helps identify the most suitable energy source for specific applications.

RF energy harvesting suits modern applications like Industry 5.0, IoT, biomedical devices, and wearables. It delivers continuous, reliable power to sensors and communication devices in smart factories, reducing maintenance and increasing flexibility [20]. IoT devices maintain performance with minimal battery replacements [21]. Biomedical implants use RF energy harvesting to improve patient comfort and device reliability [22]. Wearables benefit from compact, lightweight power sources by utilizing available RF signals [22,23]. RF energy harvesting also applies to smart homes, environmental monitoring, and agricultural sensors, expanding its range of uses [24].

### 2.1. History of Wireless Power Transfer

Hans Christian Oersted discovered the relationship between electric currents and magnetic fields in 1819, revealing that electric currents generate magnetic fields. This discovery laid the foundation for future developments in electromagnetism and modern technologies [25]. Oersted’s findings contributed to key theoretical advancements, including Ampere’s Law and Faraday’s Law.

Building on these principles, James Clerk Maxwell introduced Maxwell’s Equations in 1864, greatly enhancing the understanding of the interaction between electric and magnetic fields [26]. His 1873 book highlighted the interconnected nature of these forces, setting the stage for further technological developments, including wireless power transfer (WPT).

In 1888, Heinrich Hertz provided the first empirical proof of electromagnetic waves, using advanced instruments to transmit electricity over short distances. This marked a significant milestone in electromagnetic wave research [27]. Inspired by Hertz, Nikola Tesla developed alternating current (AC) systems that revolutionised electricity distribution. Tesla’s work, including his solutions to coil overheating, laid the groundwork for modern WPT technology [28]. His famous 1899 Tesla coil experiment demonstrated the feasibility of wireless energy transmission [29].

Tesla’s subsequent experiments, conducted between 1899 and 1901, aimed to transmit electrical energy wirelessly over long distances. However, the limitations of available technology constrained these efforts. A major breakthrough in WPT occurred in 1964 when W.C. Brown successfully powered a helicopter using microwaves. Later, in 1975, the JPL Goldstone Facility transmitted 450 kW of power through a 26-meter antenna, converting it to 30 kW of DC power using a rectenna. Innovations in microwave technology and magnetron development during World War II significantly contributed to these advancements [30].

The 1970s and 1980s witnessed further progress in microwave-powered systems, with notable contributions from researchers in Japan and Canada. NASA’s 2000 SERT program and the 2007 demonstration of near-field power transfer by Marin Soljacic at MIT were further milestones in WPT development [31]. The establishment of the Wireless Power Consortium in 2008 and the creation of the “Qi” standard in 2010 marked significant progress in the standardisation of wireless charging technologies, as demonstrated by commercial products like the Mophie 3-in-1 charger [32].

**Figure 2:**
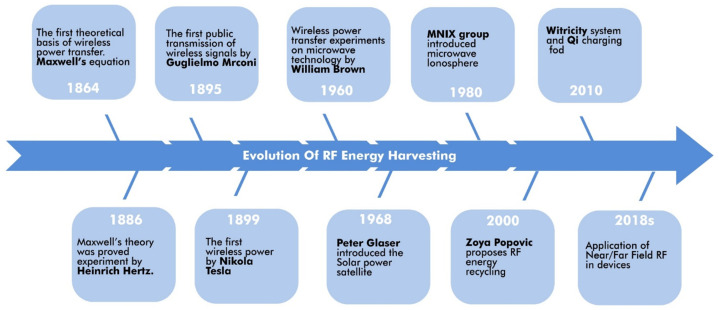
Milestones of WPT.

Figure 2 illustrates key milestones in the evolution of wireless power transfer (WPT), categorizing it into two primary approaches: non-radiative (near-field) and radiative (far-field), as outlined in Table 2. Non-radiative methods, such as inductive and capacitive coupling, rely on electromagnetic field coupling to transfer power over short distances. Inductive coupling is widely used in modern wireless charging systems, such as those based on the Qi standard, while capacitive coupling is employed in specific low-power applications. In contrast, radiative methods use radio frequency (RF) principles, transmitting power over longer distances through RF waves or laser beams.

Radiative (far-field) methods are particularly important for long-distance power transmission, such as space-based solar power or remote sensing applications, where energy needs to be transferred over significant distances with minimal infrastructure. RF waves and microwave technologies play a critical role in enabling these systems, as they can transmit power efficiently over large distances.

Despite the potential of far-field WPT, several practical challenges remain. Energy losses due to atmospheric interference, scattering, and absorption reduce the efficiency of RF and microwave power transmission over long distances. Safety concerns related to high-power RF waves and lasers pose additional challenges, particularly in ensuring that the transmission of power does not interfere with populated areas or sensitive equipment. Regulatory issues surrounding the allocation of frequencies for RF-based WPT further complicate large-scale implementations.

Nevertheless, far-field WPT holds significant potential for advanced applications such as wireless sensors, other IoT devices, where energy harvested in space can be transmitted back to Earth. Addressing efficiency losses, safety concerns, and regulatory hurdles will be critical to unlocking the full potential of far-field WPT for the future of energy transmission.

### 2.2. Technical Advancements and Challenges in Far-Field RF Energy Harvesting and Wireless Power Transfer

In RF wireless power transmission (WPT), electrical energy is converted to RF energy, transmitted through free space, and reconverted into electrical energy at the receiver. The transmission distance determines whether the signal operates in near-field or far-field scenarios, defined by the Fraunhofer distance. Far-field WPT involves radiative energy transfer using electromagnetic waves, enabling energy transmission over long distances. Wireless energy harvesting (WEH) in far-field applications captures RF energy from ambient or dedicated RF transmitters [35].

**Figure 3:**
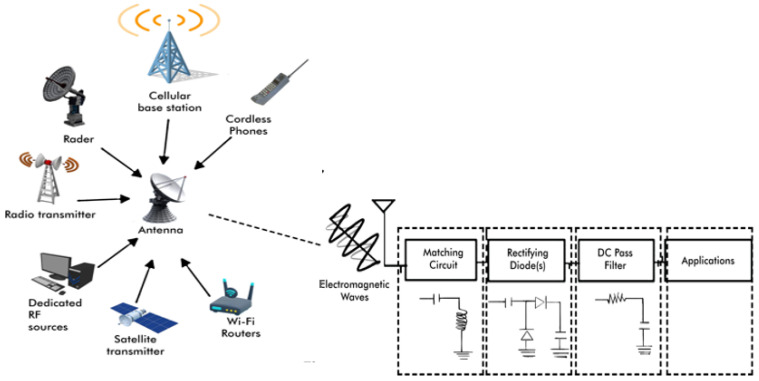
RF energy harvesting architecture.

Far-field RF energy harvesting systems use antennas to capture RF signals from ambient sources such as television and radio broadcasts, mobile phones, base stations, and wireless networks, as well as dedicated RF sources. The system architecture, shown in Figure 3, consists of a receiving antenna, an impedance matching network to optimize power transfer, and an RF-DC rectifier that converts RF energy into DC power. This energy powers low-power devices like wearables, medical implants, and sensors. Antennas can be single-band, multiband, or broadband, allowing reception from multiple frequency bands. ISM bands, often used for dedicated RF energy transfer, are regulated by the FCC to ensure safe transmission levels [36]. The Friis transmission equation estimates the received power in a far-field WPT system, considering transmitted and received power, antenna gains, wavelength, and distance. However, it provides only an approximation, as real-world factors like environmental attenuation and signal variability are not accounted for [37].

(1) $$M_{r}=M_{t}F_{t}F_{r}{\left(\frac{\lambda }{4\pi R}\right)}^{2}$$

where $M_{r}$ represents received power, $M_{t}$ is transmitted power, $F_{t}$ and $F_{r}$ are transmitting and receiving antenna gains, λ is wavelength, and *R* is the distance from the transmitting antenna. RF sources are classified as either dedicated or ambient. Dedicated RF sources transmit energy using ISM bands and regulated frequencies, ensuring safe power limits. Ambient RF sources, such as TV and radio towers, offer free energy, but their variability can make energy harvesting less predictable [36,38].

Certain frequency bands are commonly used for far-field RF energy harvesting, including microwave frequency, UHF (300 MHz to 3 GHz), L Band (1 GHz to 2 GHz), and S-Band (2 GHz to 4 GHz). The efficiency of RF energy harvesting systems depends heavily on the design of key components like impedance matching circuits and RF-DC rectifiers, which maximize power transfer and conversion efficiency.

Long-distance RF energy harvesting is constrained by the inverse-square law, which reduces power density as the distance from the source increases. High-gain antennas are necessary for capturing sufficient energy over long distances. Wideband antennas can collect energy across multiple frequencies, while multiband antennas reduce transmission losses in specific bands. Antenna performance is influenced by gain, bandwidth, and polarization. Omnidirectional antennas are useful for capturing signals from various directions, while unidirectional antennas focus on long-range transmission. Circularly polarized antennas capture signals with varying polarization, but optimizing bandwidth remains key to improving RF power recovery, as ultra-wideband antennas can sometimes reduce efficiency.

Environmental factors, such as atmospheric interference, obstacles, and urban settings, further complicate long-distance RF energy transmission. For example, in dense urban areas, multipath interference can degrade signals due to reflections from buildings. Additionally, atmospheric conditions like humidity and temperature fluctuations attenuate signals, reducing the energy that can be harvested. Developing adaptive antennas and systems that adjust to these conditions will be crucial for improving far-field WPT efficiency.

Despite these challenges, advances in high-gain antenna designs and impedance matching circuits are pushing the boundaries of far-field WPT. These innovations show promise for applications like sensors; space-based solar power, where energy captured in space could be transmitted back to Earth; or for powering IoT networks in remote areas where traditional power infrastructure is unavailable. Continued research to address efficiency losses, safety concerns, and regulatory constraints will be essential to unlocking the full potential of far-field WPT.

Far-field RF energy harvesting also relies on various antenna designs, including linear wire antennas [39], loop antennas [40], array antennas [41,42], microstrip antennas [43], and aperture antennas [44], each contributing to optimizing power transfer over long distances.

## 3. Antennas Design for RF Energy Harvesting System

Radio frequency (RF) energy harvesting relies on antennas to convert electrical signals into electromagnetic waves and vice versa. Devices like TVs, radios, cell phones, and satellite systems depend on antennas, which vary in design based on frequency, application, and signal characteristics. Impedance matching is a key challenge in rectenna design to maximize power transfer and minimize signal reflections. Antenna gain, bandwidth, and polarization are critical for optimal performance in wireless power transfer (WPT) and RF energy harvesting (RFEH). While high-gain antennas are useful for RF energy harvesting, increasing directivity does not always improve energy capture. The right balance of gain and bandwidth is essential for efficient power recovery from multiple sources. Wideband antennas capture power across multiple frequencies, and multiband antennas generate power more efficiently, avoiding route loss [45].

Antenna gain measures efficiency in transmitting or receiving power in a specific direction. High-gain antennas are essential for RF energy harvesting but are most effective when the energy source is well-known. In some cases, moderate gain suffices if the antenna is well designed [46]. The effectiveness of antennas depends on their pattern, shape, beamwidth, and polarization. Omnidirectional antennas are ideal when the direction of incoming waves is uncertain, while unidirectional antennas suit long-distance transmission. Circularly polarized antennas capture energy from multiple polarizations, while dual linearly polarized antennas reduce polarization mismatch for improved reception [45].

In RF energy harvesting, dipole antennas are widely used for basic rectenna applications, providing broad frequency response [47,48]. Microstrip patch antennas are popular for their low profile and easy fabrication, achieving circular polarization for enhanced energy conversion [49,50]. Monopole antennas, often used in mobile communications and RFID systems, are adaptable for circular polarization [51,52].

For broadband coverage and circular polarization, spiral antennas are effective in radar and wideband communications [53,54]. Yagi-Uda antennas provide high gain and directionality, making them suitable for point-to-point communications and targeted energy harvesting [55,56]. Horn antennas, common in microwave and satellite communications, support circular polarization and are efficient for high-frequency energy harvesting [54,57]. Slot antennas are compact and suitable for RFID and integrated circuits [58,59]. Parabolic reflector antennas, paired with circularly polarized feed horns, offer extremely high gain for long-range wireless power transfer and satellite communications [60].

Recent advancements include a multiband microstrip patch antenna optimized for LoRa WAN and cellular frequencies, developed using the Coyote Optimization Algorithm, achieving a peak gain of 3.94 dBi [49]. A dual-band receiver antenna developed for energy harvesting at 2.4 GHz and 5.8 GHz reached RF-DC conversion efficiencies of 63% and 54.8% [61].

For compact devices, a small antenna utilizing GSM-900, UTMS2100, and TD-LTE bands was developed to address miniaturization challenges [62]. Multiband and broadband antennas, such as self-complementary slot and patch designs, have been developed to improve RF energy harvesting efficiency by matching rectifier impedance at 2.45 GHz [63]. A triple-band differential antenna targeting frequencies from 2.1 GHz to 3.8 GHz reached maximum efficiencies of 53% and over 59% efficiency at −10 dBm in RF-DC conversion [64,65]. Fractal-based antennas have shown up to 78% RF-to-DC conversion efficiency, making them ideal for compact energy harvesting systems [66].

Miniaturization remains a key challenge for mobile and wearable devices, where small form factors are critical. While high-gain antennas are effective for RF energy harvesting, their size can limit their use in portable systems. Environmental factors such as signal interference and atmospheric conditions also impact energy harvesting efficiency, especially in dense urban environments where multipath interference is common. Designing antennas to adapt to these conditions is crucial for improving long-distance power transmission. Additionally, manufacturing cost is a concern for scaling high-performance antennas for larger applications like IoT networks and wireless sensor systems. Table 3 contains a detailed summary of various multiband and broadband antenna designs for rectenna applications. These antenna designs target different frequency bands and provide high RF-to-DC conversion efficiencies for specific applications, contributing to more efficient energy harvesting systems.

### 3.1. Antennas Arrays for Rectenna Design

In RF energy harvesting (RFEH) and wireless power transfer (WPT), antenna arrays outperform single rectennas for higher power needs by improving gain, beam steering, and interference suppression. Their performance depends on element spacing, excitation phase, and amplitude, with larger arrays boosting DC combiner efficiency [47]. Recent designs like printed Yagi [55], square patch [68], and dielectric resonator arrays [69] aim to maximize RF capture. Arrays reduce coupling effects and widen beamwidths, improving efficiency [70]. Some arrays direct RF power to a single rectifier, while others use separate rectifiers for each antenna. Despite advancements, rectifier integration remains a challenge [71]. Ferrite cores and multi-element designs mitigate coupling and maintain multiband performance. Arrays with baffles and reflectors cover 2G, 3G, and 4G bands, optimizing gain and cross-polarization [72].

A Quasi-Yagi array, designed on a Rogers 5880 substrate (0.762 mm thick, relative permittivity 2.2), operates at 2.3–2.63 GHz and is optimized for RFEH. Its compact size (26 mm by 190.5 mm) achieves a peak gain of 8.7 dBi and 25% RF-to-DC conversion efficiency at 2.45 GHz [55]. A stacked microstrip patch array, designed on a 1.6 mm FR4 substrate (relative permittivity 4.4), operates between 3.3 and 3.9 GHz, with a peak gain of 7.8 dBi at 3.5 GHz [68].

The MIMO rectangular patch array, designed on a 1.6 mm FR4 substrate, operates at 2.32 GHz and 2.8 GHz. Despite its relatively low gain (−10.13 dBi), the MIMO configuration improves signal reception and diversity [70]. A dipole antenna array on a Rogers 4350 substrate (0.8 mm thick, permittivity 3.48) operates over 0.76–0.88 GHz, 1.9–2.7 GHz, and 3.3–3.9 GHz. With gains of 6.9 dBi at 0.76 GHz and up to 10.6 dBi at 3.6 GHz, it achieves efficiencies of 57%, 49.5%, and 60.44%, respectively [72].

An antenna system with reflectors and subarrays, designed on a ferrite-loaded substrate (1.0 mm thick, permittivity 15, permeability 1000), operates from 1.7 to 2.7 GHz. It covers 380 mm by 350 mm, with a peak gain of 8.9 dBi [73]. A unidirectional four-patch array, designed on a Rogers 4350 substrate (0.8 mm thick, permittivity 3.48), operates at 2.45 GHz, achieving a peak gain of 12.7 dBi and 81.5% efficiency [74].

A two-element lower band and five-element upper band array on an FR4 substrate operates at 0.69–0.96 GHz and 1.7–2.7 GHz. This array measures 43 mm by 43 mm and provides a peak gain of 14.65 dBi with 35% and 45% efficiency across the bands [75]. A square patch array, providing omnidirectional coverage between 1.65 and 2.76 GHz, achieves a peak gain of 8.5 dBi and 22% efficiency at 2.45 GHz [76]. A twelve-element Vivaldi array, built on a Rogers RT6002 substrate (0.5 mm thick, permittivity 6.15), operates at 1.7–1.8 GHz and 2.1–2.7 GHz, achieving peak gains of up to 4.33 dBi and efficiency above 60% [77].

Table 4 compares various antenna arrays designed for rectenna applications, detailing their substrates, frequency ranges, dimensions, gains, and efficiencies.

### 3.2. Enhancing Rectenna Antenna Performance with Metamaterials Design

Metamaterials, characterized by their engineered electromagnetic properties such as electric permittivity (ε) and magnetic permeability (μ), significantly improve antenna functionality. Substrates with negative permeability and permittivity are classified as double-negative (DNG) materials, while those with positive values for both properties are double-positive (DPG). Mu-negative (MNG) materials exhibit negative permeability, and epsilon-positive (EPG) materials show positive permittivity. Single-negative (SNG) materials exhibit negative permittivity or permeability, depending on the specific electromagnetic property [78]. Integrating metamaterials into antenna design enhances performance in RF energy harvesting systems. These materials improve antenna gain and efficiency, facilitating better capture of ambient RF energy. Metamaterials also enable the development of compact antennas with optimized radiation patterns, making them ideal for energy harvesting from specific directions. Their adaptability to varying RF environments enhances antenna effectiveness.

Maxwell’s first-order differential equations form the foundation of electromagnetism [79]. The conservation law is written as a continuity equation:

(2) $$I=\oint_{A}J\cdot ds=\;\frac{\partial }{\partial t}\int_{V}\rho \;d\tau$$

In this equation, ω represents angular frequency, H is the magnetic field, E is the electric field, B denotes magnetic flux density, J is current density, and ρ is charge density. Applying the divergence theorem leads to the differential form of the continuity equation:

(3) $$\nabla \cdot S=-\frac{d\rho }{dt}$$

Starting from Ampere’s law in differential form:

(4) $$\nabla \times B=\mu_{0}J$$

Taking the divergence of both sides:

(5) $$\nabla \cdot \left(\nabla \times B\right)=\mu_{0}\nabla \cdot J=0$$

If there are time-varying charge densities, ∇·J=0 is not compatible with the continuity equation. Only in the electrostatic limit is Ampere’s law accurate. The addition of displacement current to the RHS provides a more complete solution.

(6) $$\nabla \times B=\mu_{0}(J_{D}+J),\;\nabla \cdot J_{D}=\frac{d\rho }{dt}$$

In the formula for the displacement current, we may apply Gauss’ Law to change from ρ to ∇·E:

(7) $$\nabla \cdot J_{D}=\epsilon_{0}\frac{d(\nabla \cdot E)}{dt}$$

Eliminating the divergencies:

(8) $$J_{D}=\epsilon_{0}\frac{dE}{dt}$$

The displacement current is the electric field’s time derivative. Ampere’s law, modified with displacement current:

(9) $$\nabla \times B=\mu_{0}\left(\epsilon_{0}\frac{dE}{dt}+J\right)$$

Therefore, the four differential equations below, sometimes referred to as Maxwell’s equations, provide an overview of the rules of electromagnetism:

(10) $$\begin{array}{cccc} \;\nabla \cdot E & =-\frac{\rho }{\epsilon_{0}} & & \;\mathrm{Gauss}’\;\mathrm{law}\;\mathrm{to}\;\mathrm{determine}\;E \end{array}$$

(11) $$\begin{array}{cccc} \;\nabla \cdot B & =0 & \; & \;\mathrm{Gauss}’\;\mathrm{law}\;\mathrm{to}\;\mathrm{determine}\;B\; \end{array}$$

(12) $$\begin{array}{cccc} \;\nabla \times E & =-\frac{dB}{dt} & \; & \;\mathrm{Faraday}’s\;\mathrm{law}\;\mathrm{of}\;\mathrm{Induction} \end{array}$$

(13) $$\begin{array}{cccc} \nabla \times B & =\mu_{0}\left(\epsilon_{0}\frac{dE}{dt}+J\right) & \; & \mathrm{Modified}\;\mathrm{Ampere}’s\;\mathrm{law} \end{array}$$

The electric and magnetic fields for plane waves are determined by:

(14) $$\begin{array}{cc} E & =\;E_{0}e^{-(ik\cdot r\mp i\omega t)} \end{array}$$

(15) $$\begin{array}{cc} H & =\;H_{0}e^{-(ik\cdot r\mp i\omega t)} \end{array}$$

where r is the distance, and k is a wave vector that may be represented in terms of the orthogonal system’s electric and magnetic fields. The flow of energy is determined by the real portion of the Poynting vector:

(16) $$S_{1}=0.5E\times H$$

Changing the sign of $\mu_{0}$ and $\epsilon_{0}$ has no effect on the direction of energy flow. As a result, the group velocity is positive for both left- and right-handed orthogonal systems, and it is given as:

(17) $$n=\pm \sqrt{\epsilon_{0}\mu_{0}}$$

The phase velocity ($V_{m}$) is expressed as:

(18) $$V_{m}=\frac{C}{n}$$

In this equation, $V_{m}$ represents the phase velocity, *n* is the refractive index, and *C* is the speed of light. Forward wave motion results from the fact that energy and waves propagate in the same direction in a right-handed system. Conversely, a left-handed system exhibits a negative value of *n*, resulting in a corresponding negative phase velocity ($V_{P}$) and retrograde propagation of the waves. This reverse propagation increases the reflected wave’s intensity.

By virtue of their distinct characteristics, substances possessing negative electric permittivity (ε) and positive permeability (μ) are classified as electric plasmas. Surface plasmon generation necessitates the combination of a structured metallic strip, a dielectric coupler, and surface irregularity. Thin metallic filaments that are arranged periodically in a dielectric medium are capable of exhibiting low-frequency stopband characteristics. Rotman investigated diverse wire network configurations capable of producing a controlled negative electric permittivity by resonating with the polarized electric field at frequencies below the plasma frequency. The utilization of a designated formula enables the computation of the effective plasma frequency in this manner [80]:

(19) $$\omega^{2}=\frac{2\pi c_{0}^{2}}{a^{2}\left[\ln\left(\frac{a^{2}}{4r(a-r)}\right)\right]}$$

Essentially magnetic plasmas, negative materials have positive permittivity (ε) and negative permeability (μ). Their magnetic response at optical frequencies is low due to a weak interaction with light’s magnetic field, similar to the Bohr magneton. Metallic staples, split-ring resonators (SRRs), nanorods, nanoplates, and strips exhibit negative permeability magnetic responses at high frequencies. The SRR, an inductor–capacitor design, is the most common negative permeability structure. Pendry’s sub-wavelength structures have opposing concentric split-ring resonators. Capacitive metal rings collect charges, although circular current cannot travel through their voids.

Analytical formulas can calculate inductance, capacitance, and resonant frequency for complementary split-ring resonators (CSRRs), loop gaps, and SRRs. Spirals, Swiss rolls, squares, and hexagons can also cause negative permeability. Adjusting the geometric characteristics of single and double split-ring resonators can shift SRR resonant frequencies across a wide range. Compactness and tight packing make rectangular SRRs better than circular ones.

The overall capacitance of an SRR circuit is the sum of its upper and lower capacitances divided by the ring gap line. The circuit’s resonance frequency can be calculated using the following equation [81]:

(20) $$\omega_{pf}=\sqrt{\frac{2}{\pi r_{1}LC_{p}}}$$

where $C_{p}$ is the per-unit-length capacitance between the rings, *L* is the total inductance of the SRR under consideration, and $r_{1}$ is the average radius of the SRR under consideration.

**Figure 4:**
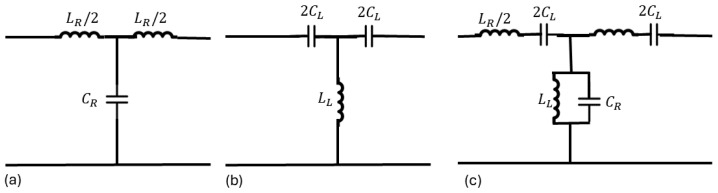
Circuit models for transmission lines in composite right/left-hand metamaterials.

Left-handed materials (LHMs), exhibiting both left-handed and right-handed characteristics, are implemented through transmission lines and resonators, specifically as composite right/left-hand metamaterials (CRLHs). The CRLH Transmission Line (CRLH-TL) method uses circuit models to analyze these materials, including three transmission line types: right-handed (RH), left-handed (LH), and CRLH lossless lines. Figure 4a depicts the right-handed transmission line (RH-TL) circuit model, consisting of a series inductor $L_{R}$ and a shunt capacitor $C_{R}$. In this configuration, the right-handed behavior is characterized by positive phase velocity, where energy propagates in the same direction as the wave vector, corresponding to traditional forward-wave transmission. The RH-TL model effectively represents conventional wave propagation and is typically employed in higher-frequency applications. However, it does not support backward-wave propagation, as it is limited to right-handed properties.

Figure 4b shows the left-handed transmission line (LH-TL) circuit model, which is the dual of the RH-TL. It features a shunt inductor $L_{L}$ and a series capacitor $C_{L}$, enabling backward-wave propagation, where the phase velocity is negative. In this model, energy flows in the opposite direction to the wave vector, a phenomenon specific to left-handed materials. Despite its theoretical potential, the practical implementation of purely left-handed structures is hindered by parasitic effects, which cause deviations from ideal performance, particularly at higher frequencies. Figure 4c illustrates the circuit model of a composite right/left-handed transmission line (CRLH-TL), which integrates both right-handed elements (series inductor $L_{R}$ and shunt capacitor $C_{R}$) and left-handed elements (shunt inductor $L_{L}$ and series capacitor $C_{L}$). The CRLH-TL structure offers a solution to the limitations of traditional LH-TL designs by supporting dual-band operation, with backward-wave propagation at lower frequencies due to the LH components and forward-wave propagation at higher frequencies due to the RH components. This model represents a generalized CRLH-TL, where the continuous transition between left-handed and right-handed behavior occurs at the transition frequency, where the phase velocity crosses zero. By mitigating the parasitic effects that affect LH-TLs, the CRLH-TL model enhances the practicality and applicability of transmission line metamaterials in modern technology.

A transmission line’s (TL) propagation constant (ρ) is calculated as follows:

(21) $$\rho =\alpha +j\gamma =\sqrt{YZ}$$

where *Y* and *Z* denote admittance and impedance, respectively. Using the CRLH-TL, *Z* and *Y* can be calculated as follows [82]:

(22) $$Z\left(\omega \right)=j\left(\omega L_{R1}-\frac{1}{\omega C_{L1}}\right)$$

(23) $$Y\left(\omega \right)=j\left(\omega C_{R1}-\frac{1}{\omega L_{L1}}\right)$$

Therefore, a homogeneous CRLH will exhibit the dispersion relation shown below:

(24) $$\gamma \left(\omega \right)=k\left(\omega \right)\sqrt{\omega^{2}L_{R1}C_{R1}+\frac{1}{\omega^{2}L_{L1}C_{L1}}-\left(\frac{L_{R1}}{L_{L1}}+\frac{C_{R1}}{C_{L1}}\right)}$$

where the wavenumber k(ω) can be determined by:

(25) $$k\left(\omega \right)=\left\{\begin{array}{cc} -1 & \mathrm{if}\;\omega <\omega_{\iota 1}=\min\left(\frac{1}{\sqrt{L_{R1}C_{L1}}},\frac{1}{\sqrt{L_{L1}C_{R1}}}\right)\\ +1 & \mathrm{if}\;\omega <\omega_{\iota 2}=\max\left(\frac{1}{\sqrt{L_{R1}C_{L1}}},\frac{1}{\sqrt{L_{L1}C_{R1}}}\right) \end{array}\right.$$

The phase constant can be real or imaginary, depending on the sign of the radicand. An imaginary phase constant corresponds to a passband where wave propagation occurs, while a real phase constant indicates a stopband where propagation is blocked. CRLH-TL exhibits this stopband behavior, unlike RH-TL and LH-TL. Analyzing LC networks might use SMT chip components or discrete components, but SMT-based CRLHs are limited to lower frequencies and are not suitable for radiating applications. CRLH designs can be applied using CPW, stripline, or microstrip transmission lines [80].

Metamaterial antenna design methods include metamaterial loading, which uses negative ε/μ materials and high permeability shells; metamaterial-inspired antennas, such as SRRs and CSRRs; metasurface loading with EBG, RIS, HIS, and FSS technologies; and composite right/left-hand materials, featuring zeroth-order (ZOR) and first resonators [83].

#### 3.2.1. Miniaturization of Antennas Using Metamaterial-Inspired Design

Researchers have made significant strides in miniaturizing antennas by integrating metamaterials such as mu-negative (MNG), epsilon-negative (ENG), and double-negative (DNG) materials into their designs. These materials, known for their engineered electromagnetic properties, allow antennas to maintain or improve performance while reducing size. Metamaterials achieve this by inducing resonant behaviors and generating in-phase currents, which improve radiation efficiency and enable compact designs. Additionally, RLC resonant structures and split-ring resonator (SRR/CSRR) cells help fine-tune antenna characteristics, further optimizing size and performance. The use of metasurfaces and Frequency Selective Surfaces (FSSs) with composite right/left-hand (CRLH) unit cells enhances antenna performance by modifying the electromagnetic field distribution around the antenna, thus reducing physical dimensions without sacrificing gain or bandwidth [81].

The mechanics of miniaturization using metamaterials are primarily based on their ability to alter the effective wavelength of the electromagnetic waves interacting with the antenna. In a conventional antenna, the size is typically related to the wavelength of operation, with longer wavelengths requiring larger antennas. Metamaterials, by introducing negative permittivity (ε) or permeability (µ), effectively compress the wavelength within the material, allowing antennas to operate at the same frequency while being physically smaller. This is particularly advantageous for consumer electronics, where space is limited, and compact, efficient antennas are critical.

For instance, a compact, dual-band metamaterial antenna designed for body-centric wireless communication achieves miniaturization through the use of a zeroth-order loop for omnidirectional radiation and a circular patch for unidirectional patterns, allowing it to support both on- and off-body communications. The use of metamaterials to increase substrate permeability allows for the combination of a quarter-wave shorted patch antenna with a T-shaped probe dipole, significantly reducing the antenna’s size without compromising performance. This design, which includes a modified split-ring resonator (MSRR) on a Rogers 5870 substrate (0.762 mm thick with a permittivity of 2.33), operates within the 1.7–2.67 GHz frequency range. The antenna measures $0.55\lambda_{0}\times 0.363\lambda_{0}$, delivering a peak gain of 8.51 dBi and an efficiency of 45% [84,85].

Another example involves a complementary split-ring resonator (CSRR) antenna designed on a Rogers RT5800 substrate sourced from Rogers Corporation, located in Chandler, AZ, USA (1.0 mm thick, permittivity of 2.2). Operating at 1.8 GHz, this antenna has a footprint of 63.8×83.8 mm^2^ and achieves a peak gain of 3.1 dBi [86]. The inclusion of SRR/CSRR structures in the antenna design allows for a concentrated electric field in a small area, thereby reducing the physical size of the antenna while maintaining effective radiation characteristics.

Another design incorporates a metasurface with capacitive loading, built on a Rogers 4003C substrate (0.81 mm thick, permittivity of 3.55). This metasurface operates across the 3.02–3.63 GHz frequency range. It achieves a peak gain of 6.57 dBi and has compact dimensions of $0.58\lambda_{0}\times 0.58\lambda_{0}\times 0.043\lambda_{0}$. The metamaterial-based design enables the antenna to miniaturize while maintaining high performance, with efficient radiation patterns suitable for various wireless applications [87].

Metamaterials are particularly useful in dual-band antennas, as demonstrated in a GPS antenna array with broadside coupled split-ring resonators (BC-SRRs) and mu-negative (MNG) metamaterials. This design reduces mutual coupling in the L band, which enhances performance and reduces the antenna footprint. The use of metamaterials in this context not only improves the radiation efficiency but also significantly shrinks the overall size of the antenna, making it more suitable for compact systems [88].

In summary, the integration of metamaterials into antenna design enables significant size reduction by altering the propagation characteristics of electromagnetic waves. By compressing the effective wavelength within the metamaterial, antennas can be miniaturized without losing efficiency or performance. This innovation is crucial for developing compact antennas for wireless energy harvesting, wearable devices, and IoT applications, where space constraints and performance demands are high.

Table 5 provides a summary of the various metamaterial-inspired miniaturization methods, detailing the designs, materials used, frequency ranges, gains, and the corresponding size reductions achieved through these techniques.

#### 3.2.2. Bandwidth and Gain Enhancement Antenna Using Metamaterial-Inspired Designs

Metamaterials significantly improve antenna performance by enhancing both bandwidth and gain. Bandwidth enhancement is achieved through techniques such as using thick, low-permittivity substrates, multi-layer designs, and advanced geometries. A thicker substrate increases the storage and radiation of electromagnetic energy, thereby widening the bandwidth. For example, a thick substrate provided a bandwidth of 58.8% (2.11–3.87 GHz) while maintaining omnidirectional radiation and low cross-polarization [89]. Multi-layer structures and innovative geometries, such as fractal designs, allow the antenna to operate across a wider frequency spectrum by supporting multiple resonances.

Gain enhancement is achieved by employing metamaterial-based designs, such as stacked microstrip patches, fractal geometries, and gap-coupling techniques. These innovations focus more electromagnetic energy in a specific direction, increasing efficiency and reducing back radiation. Stacked microstrip patches expand the effective radiating area of the antenna without increasing its physical size, while fractal geometries enable multi-frequency resonance by exploiting their self-similar structure. Gap-coupling improves impedance matching between the antenna and the transmission line, minimizing reflection losses and further boosting gain. Additionally, metamaterial lenses and artificial magnetic conductors (AMCs) enhance gain by shaping and focusing the electromagnetic field, which is particularly valuable for low-frequency antennas.

In low-frequency applications, unidirectional loop antennas integrated with mu-negative (MNG) metamaterials and symmetric split-ring resonators (SSRRs) deliver consistent gain and wide bandwidth by carefully controlling the electromagnetic field distribution, thereby minimizing energy losses. Slotless ground planes and near-zero index metamaterial structures (NZIMSs) further improve performance by optimizing directivity and reducing the antenna’s physical footprint. NZIMSs work by altering the wave’s phase velocity, which makes it easier to achieve high-gain, narrow-beamwidth characteristics in a compact design [68].

Recent advancements in metamaterial-based antenna designs have demonstrated significant improvements in key performance metrics. A prime example is the zeroth-order resonator (ZOR) antenna, which employs a Rogers RO4003C substrate (0.81 mm thick, permittivity 3.55). Operating between 348 and 772 MHz, the ZOR antenna achieves a bandwidth of 78% and a gain of 9.2 dBi. Its compact dimensions of $0.46\lambda_{0}\times 0.46\lambda_{0}$ make it highly effective for low-frequency applications, where large antenna sizes are typically required [90]. The ZOR design takes advantage of metamaterial properties to reduce the effective wavelength within the structure, enabling resonance with a much smaller physical size.

Another design incorporates split-ring resonators (SRRs) on an FR4 substrate (1.6 mm thick, permittivity 4.4). Covering a wide frequency range from 0.865–1.06 GHz to 4.9–6.5 GHz, this multiband antenna achieves a gain of 6.74 dBi, with compact dimensions of 78.6 × 42.5 mm^2^. This versatility makes it well-suited for various energy harvesting applications [91]. The SRR structures enable efficient operation over multiple frequency bands by creating strong localized resonances, improving the antenna’s multiband performance.

A metamaterial-loaded antenna, constructed on a Rogers 6010 substrate (0.635 mm thick, permittivity 6.15), operates at 0.915 GHz and 2.45 GHz, offering bandwidths of 17.8% and 35.8%, respectively. With gains of 17.8 dBi and 9.81 dBi, the compact dimensions of 7×6×0.254 mm³ highlight the effectiveness of metamaterials in boosting both bandwidth and gain [92]. The metamaterial loading improves energy concentration, ensuring high gain despite the compact antenna size.

Lastly, an antenna employing near-zero-index metamaterials on an FR4 substrate (1.6 mm thick, permittivity 4.6) operates at 0.534 GHz, offering a bandwidth of 2.11% and an efficiency of 74.1%, with a gain of 7.27 dBi and dimensions of 170 × 170 mm^2^. NZIMSs enhance the effective aperture size without increasing the physical footprint, making this design particularly effective for low-frequency applications, where large antenna sizes typically limit performance [93].

Table 6 summarizes the various bandwidth and gain enhancements achieved through metamaterial-inspired designs, demonstrating the effectiveness of these technologies in improving antenna performance across different frequency ranges and applications.

#### 3.2.3. Circular Polarization of Antenna Using Metamaterial-Inspired Design

The effectiveness of RF energy harvesting systems is closely tied to antenna polarization, which can be either linear or circular, depending on the orientation of the electric field. Circular polarization is particularly beneficial in RF systems, as it allows antennas to capture signals from multiple orientations and mitigate polarization mismatches that commonly occur in dynamic environments. This results in more stable signal reception, especially in systems prone to multipath interference or rapidly changing signal directions. Proper alignment between the transmitter and receiver antennas minimizes cross-polarization losses, which occur when the electric fields of the antennas are misaligned.

Metamaterial structures play a crucial role in enhancing circular polarization by enabling compact antenna designs with improved performance. Metamaterials allow antennas to maintain or even increase gain while reducing size and broadening bandwidth. Additionally, metamaterials help address polarization issues by mitigating Faraday rotation, which can distort the polarization of signals as they propagate through the atmosphere. By incorporating metamaterials, antennas can achieve circular polarization with improved signal strength, often increasing gain by 3 dB.

For instance, a metamaterial-loaded cavity antenna using Rogers RT6010 and Rogers 5880 substrates with thicknesses of 0.635 mm and 0.762 mm, respectively, and permittivities of 6.15 and 2.2, operates across the frequency range of 9.7 GHz to 10.27 GHz. This design achieves an efficiency of 6.0% and a bandwidth of 74.1%, with a peak gain of 14.1 dBi. The metamaterials in this configuration significantly enhance the antenna’s circular polarization performance, allowing for higher efficiency and gain [75].

An electromagnetic bandgap (EBG) structure, fabricated on an FR4 substrate with a thickness of 1.0 mm and a permittivity of 3.5, operates at two distinct frequencies: 12.5 GHz and 14.2 GHz. This structure achieves gains of 6.0 dBi and 4.3 dBi, respectively, with an overall efficiency of 47.7%. The total gain at these frequencies reaches 23.1 dBi and 24.4 dBi. The use of an EBG structure helps control electromagnetic wave propagation, improving circular polarization and making the design suitable for high-frequency applications [79]. Metamaterial elements in the EBG structure, such as split-ring resonators (SRRs), contribute to the effective manipulation of electromagnetic fields, facilitating improved polarization control.

Another design, a frequency-agile near-field resonator antenna, incorporates parasitic elements on a Rogers 5880 substrate with a thickness of 0.762 mm and a permittivity of 2.2. Operating at 1.39 GHz, this antenna achieves an efficiency of 3.92% and a gain of 5.92 dBi. The parasitic elements enable the antenna to dynamically adapt to different operational frequencies while maintaining stable circular polarization. This design underscores the effectiveness of metamaterials in creating adaptable, high-performance antennas for RF energy harvesting systems [94].

In summary, metamaterials enable significant advancements in the deployment of circularly polarized antennas by improving gain, enhancing bandwidth, and reducing size. Through the use of engineered electromagnetic properties, such as negative permittivity and negative permeability, metamaterial-based designs offer superior control over the electromagnetic field, allowing antennas to operate more efficiently in challenging RF environments. These improvements are essential for applications in wireless energy harvesting, satellite communications, and IoT systems, where polarization stability and compact designs are critical. Table 7 provides a summary of the operating frequency, bandwidth, maximum efficiency, and gain for circularly polarized metamaterial-enhanced designs, highlighting the key performance metrics achieved through these innovations.

#### 3.2.4. Metamaterial Enhancement of Isolation and Mutual Coupling Reduction in Antenna for Multi-Port Rectenna

Maintaining proper separation between components is crucial for minimizing mutual coupling in multi-port rectenna systems. Mutual coupling occurs when energy radiated from one antenna element interferes with another, leading to performance degradation through reduced efficiency, increased signal interference, and lower data rates. Metamaterials, such as Electrically Neutral Material (ENG), Magnetically Neutral Material (MNG), and Dielectrically Neutral Material (DNG), provide effective decoupling elements when integrated into antenna designs to address these issues.

For example, a Metastrip antenna, fabricated on a Rogers 5880 substrate with a thickness of 0.762 mm and a permittivity of 2.2, operates at 28 GHz. This design achieves a gain of 20.12 dBi and provides a peak gain of 8 dBi, with a bandwidth of 22%, and an efficiency of 24%. This setup demonstrates the effectiveness of Metastrip technology in high-frequency applications [60].

Other strategies to mitigate mutual coupling include using metasurface structures to reduce surface wave propagation and enhance isolation. Near-field resonator structures, such as split-ring resonators (SRRs) and complementary split-ring resonators (CSRRs), also prevent antenna coupling. Additionally, composite right/left-handed (CRLH) structures generate reverse currents, effectively reducing mutual coupling between antennas [95].

In beamforming applications, a printed antenna array with CSRRs operating at 25 GHz effectively reduces mutual coupling while preserving the array’s radiation pattern without altering the antenna profile. This design was developed using simulation tools like Ansys HFSS version 2022 R and MATLAB Version R2023a. Furthermore, integrating a metamaterial superstrate with modified CSRRs significantly suppresses coupling between microstrip phased array elements, providing a compact and easily applicable solution for performance enhancement in various high-frequency applications [96].

A CSRR with a slot, constructed on a Rogers 6010 substrate with a thickness of 0.635 mm and a permittivity of 6.15, operates at 3.6 GHz. This design achieves a gain of 27 dBi and an efficiency of 3.59%, with a bandwidth of 35% and a peak gain of 52 dBi. This configuration highlights the use of CSRRs with slot technology to enhance antenna performance characteristics, especially in terms of gain and efficiency [97].

In summary, metamaterials provide a robust solution for reducing mutual coupling in multi-port rectenna systems, improving isolation and preserving antenna performance. By integrating elements such as SRRs, CSRRs, and CRLH structures, metamaterial-enhanced designs offer improved efficiency, higher gain, and greater bandwidth, making them ideal for advanced MIMO systems in high-frequency applications. Table 8 provides a summary of mutual coupling reduction methods and how they improve multi-port rectenna performance, detailing the key metrics like gain, bandwidth, efficiency, and isolation.

## 4. Impedance Matching Network (IMN) of Antenna and Rectifier

Impedance matching is a critical area of study in virtually all fields of electronics. Effective termination is crucial for minimizing reflections and maintaining signal integrity, particularly for communication and energy harvesting applications where signal transmission is essential [98]. In an RF network, impedance mismatch causes power to be reflected back to the source from the boundary. This reflection creates a standing wave instead of transferring energy to the load [99].

Achieving optimal performance for antennas, such as improving return loss, efficiency, and gain, involves addressing impedance matching, a challenging phase in the design process. Moreover, impedance matching simplifies tuning the antenna’s frequencies faster than altering its design. The additional resonances introduced by impedance matching circuits also improve the antenna’s bandwidth [100].

In rectifiers that incorporate diodes and transistors, the impedance fluctuates with frequency due to their nonlinear nature. This variation makes it difficult to match a standard 50-ohm antenna to an RF-DC rectifier [101]. The reflection coefficient equation, described in [102], is used to represent the electrical signal reflection resulting from this impedance mismatch:

(26) $$\Upsilon =S_{11}=S_{22}=\frac{Z_{\mathrm{rect}}-Z_{\mathrm{ant}}^{*}}{Z_{\mathrm{rect}}+Z_{\mathrm{ant}}^{*}}$$

It is commonly referred to as the $S_{11}$ parameter or reflection coefficient. Here, $Z_{\mathrm{rect}}$ denotes the rectifier’s impedance, which is represented as $R_{1}+X_{1}$, and $Z_{\mathrm{ant}}$ represents the antenna impedance, expressed as $R_{2}+X_{2}$. Additionally, $Z_{\mathrm{ant}}^{*}$ is the complex conjugate of the antenna impedance. The reflection coefficient Υ is always less than or equal to 1.

In radio frequency energy harvesting (RFEH) systems, L, T, and π-matching networks [103] are commonly used impedance matching circuits. These circuits consist of inductors and capacitors arranged in L, T, and π shapes to match the load impedance at a desired frequency.

Impedance matching for antennas can also be achieved using the distributed impedance matching technique [104]. This technique involves making structural adjustments to the antenna using stubs, single- and multi-section quarter-wave transformers, tapered lines, baluns, and active components. A key advantage of the distributed impedance matching technique is that it does not require altering the geometry of the radiating structure. As a result, the radiation efficiency of the antenna remains unaffected by the matching network, thereby simplifying the design process. However, this method increases the antenna’s size, making it less ideal for practical array systems. Additionally, the extra circuitry added to the matching network may increase spurious radiation losses, reducing system efficiency.

The impedance matching network (IMN) has unique challenges due to the very low and changing levels of input power. The input to the IMN is the rectifier’s nonlinear load impedance, which varies with the power received by the antenna. Meanwhile, the antenna provides the IMN’s source impedance, which is not necessarily 50 ohms. Therefore, the IMN must be designed for a specific level of received power and may not be optimal at varying power levels [105].

## 5. Rectifier Design for Wireless Energy Scavenging/Wireless Power Transfer System

Radio frequency energy harvesting (RFEH) devices use rectifiers to convert RF signals into direct current (DC) signals. The main obstacles in RFEH technology are the low power density of RF waves and inefficient harvesting circuits. To enhance the output DC power, multiband and broadband rectifiers (BBRs) have been developed [106]. However, the performance of rectifiers can be impacted by variations in ambient electromagnetic (EM) radiation.

**Figure 5:**
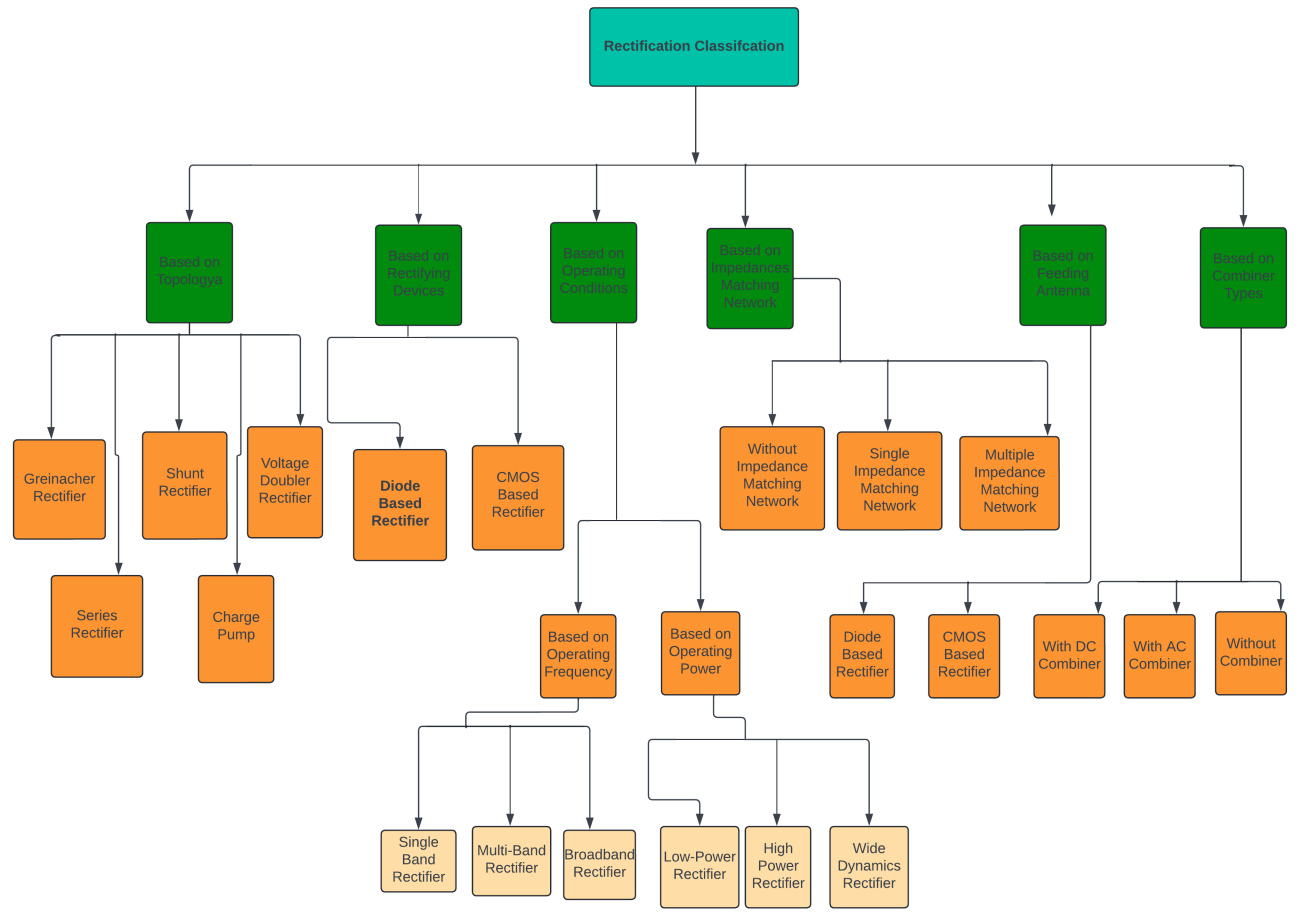
Classification of rectifiers.

Rectifier design employs various strategies to reduce the influence of input power variations. Rectifiers are categorized based on several factors, including topology, operating frequency (broadband, single band, multiband), impedance matching, type of combiner, feeding antenna, and power level (low, medium/high power, or wide input power dynamic range). As shown in Figure 5, rectifier designs vary according to their intended operational requirements.

A rectifier’s performance can be assessed using factors like sensitivity, power conversion efficiency, and power dynamic range. Sensitivity refers to the amount of input power needed to produce a 1 V DC output at the expected load. The rectifier’s effectiveness can decline as these factors change, primarily due to the nonlinearity of the Schottky diode or CMOS in the circuit. Variations in RF input power and frequency can affect the rectifier’s input impedance, causing mismatches that reduce power conversion efficiency. Ensuring consistent activation and performance of the RFEH system in practical applications depends on addressing these issues [21].

The formula for estimating the sensitivity of the rectifier is given below [107]:

(27) $$S_{\mathrm{dBm}}=10\times \log_{10}\left(P_{s}\right)$$

Rectifier sensitivity ($S_{\mathrm{dBm}}$) is in dBm, and power received ($P_{s}$) is in milliwatts (mW).

The power conversion efficiency (PCE) of a rectifier is the ratio of the DC output power to the RF input power [108]:

(28) $$P_{\mathrm{eff}}=\frac{P_{\mathrm{DC}1}}{P_{\mathrm{re}}}=\frac{\left(\frac{V_{1}^{2}}{R_{L}}\right)}{100\times \mu W}$$

where $P_{\mathrm{eff}}$ denotes the rectifier’s power conversion efficiency (PCE), $P_{\mathrm{DC}1}$ the output DC power, $R_{L}$ the load resistance, $V_{1}$ the rectifier’s output DC voltage, and $P_{\mathrm{re}}$ the RF power received by the rectifier.

The power dynamic range (PDR) of a rectifier is the input power range in which the rectifier maintains a PCE greater than 20%. This is significant because, in an RF environment with changing power densities, the greater the range, the more dependable the rectifier will be [109]:

(29) $$P_{\mathrm{DR}}=\left\{100\%\geq P_{\mathrm{eff}}\geq 20\%\right\}$$

A PDR in the rectifier is critical for ensuring the RFEH system’s dependability in an RF environment. The voltage produced from rectification is usually too low to power electronics. In RFEH systems, rectifiers are often used alongside DC–DC voltage conversion technologies like voltage multipliers, charge pumps, or voltage boosters to raise output DC voltage for electronic devices.

### 5.1. Circuit Design of CMOS Rectifier

RF rectifying transistors operate as three-terminal switches or diodes to correct signals, needing extra gate voltage. In recent CMOS rectifier designs, significant improvements have been made in performance and efficiency. A CMOS differential rectifier, operating at 2.4 GHz on a 0.508 mm thick Rogers substrate, occupies an area of 1.5 × 0.47 mm^2^ and achieves a dynamic range of 25.5 dB with an efficiency of 46% [90]. The CMOS Villard multiplier rectifier operates at both 400 MHz and 2.4 GHz, achieving an efficiency of 75% [106].

In comparison, a CMOS passive rectifier working at 0.2 GHz takes up 0.88 mm^2^ of space, offering a dynamic range from −4 to 7 dB and reaching an efficiency of 70.3% [110], while a 434 MHz differential CMOS bootstrap rectifier achieves 71% PCE [111]. RF energy harvesting technology has advanced with a cross-coupled differential-drive (CCDD) rectifier with PCE > 40%, automated design optimization using Deep Neural Networks, and a co-design approach for RF harvester matching networks and rectifiers to maximize PCE [112].

The CMOS bootstrap rectifier, operating at 0.433 GHz with a 0.30 mm^2^ area, features a dynamic range of −6 to 6 dB and achieves 71% efficiency at 3 dBm [111]. Another notable design, the cross-coupled CMOS rectifier, functions at 2.45 GHz with an area of 0.90 mm^2^ and has a dynamic range of −5 to 7 dB, achieving efficiencies of 73% at −6 dBm and 70.4% at −5.5 dBm [113].

The CMOS-CCDD rectifier, with a frequency of 0.9 GHz and unspecified area, covers a dynamic range of −21 to 13 dB and offers efficiencies of 83.7% at −18.4 dBm and 80.3% at −17 dBm [114]. The double-sided CMOS rectifier, also at 0.9 GHz, features a compact area of 0.088 mm^2^ and achieves 66% efficiency [115]. The CMOS reconfigurable rectifier operates at 0.9 GHz with a 25% efficiency [116]. Meanwhile, a CMOS rectifier functioning at 6.78 MHz covers an area of 8 mm^2^ and achieves a high efficiency of 92.2% [117].

These advancements in CMOS rectifier technology highlight a range of designs, each offering different balances of frequency, area, efficiency, and dynamic range to meet various application needs in modern electronics, as shown in Table 9.

### 5.2. Circuit Design of Schottky Diode Rectifier

A nonlinear Schottky diode is used in the rectifying circuit for RF-to-DC conversion. Understanding the impedance characteristics of this diode is crucial for designing an appropriate matching circuit. The goal is to determine the diode’s equivalent circuit model and, using small-signal analysis, understand its impedance behavior. The process for creating an effective rectifier involves selecting a suitable Schottky diode based on the available power level, and then analyzing its impedance behavior.

**Figure 6:**
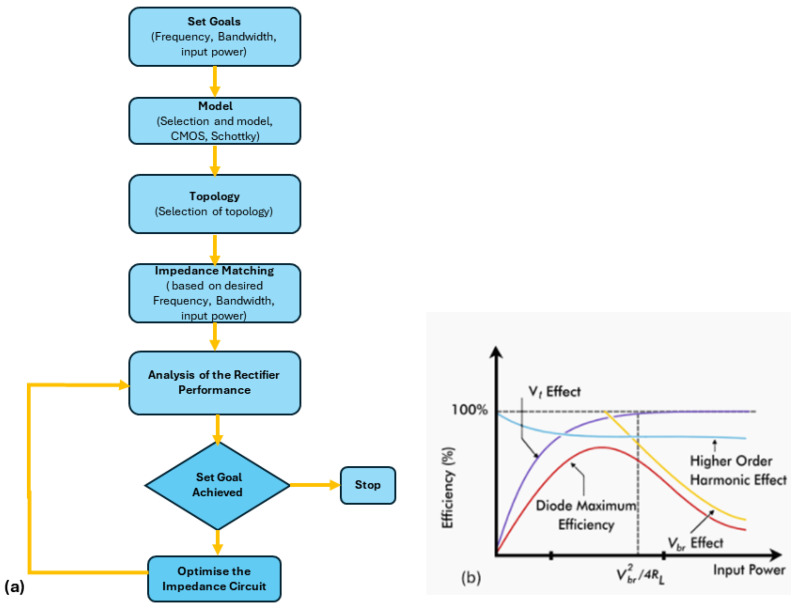
(**a**) Flowchart of rectifier design (**b**) Input power and efficiency analysis.

The design steps for an efficient rectifier are illustrated in Figure 6a. The primary determinant in choosing a diode is the available power level. The maximum diode efficiency versus input power is shown in Figure 6b. The breakdown voltage and threshold voltage of the diode limit the power handling capacity of the rectifier.

**Figure 7:**
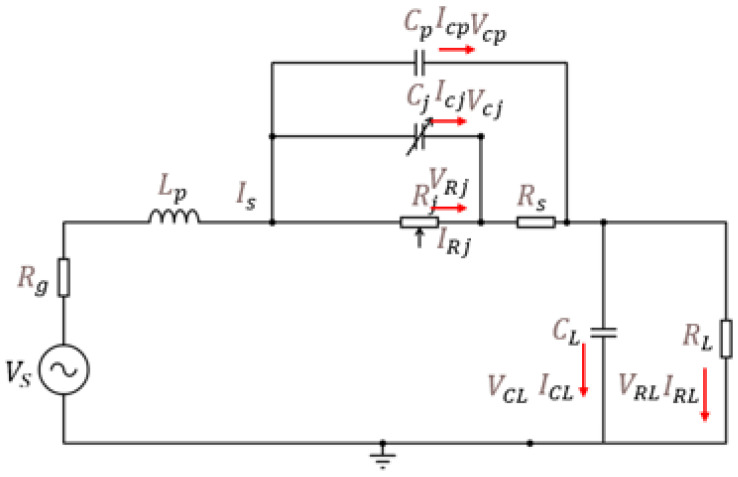
Equivalent circuit for single-diode model.

Once a suitable Schottky diode is selected, its impedance behavior is analyzed. Figure 7 shows the small-signal model of the diode, highlighting the junction resistance (Rj) and junction capacitor (Cj), which contribute to the diode’s nonlinearity. The parasitic series resistance (Rs) and inductance (Lp) are also considered.

The series resistance $R_{s}$ of the Schottky diode is given by:

(30) $$R_{s}=\frac{nkT}{q(I_{s1}+I_{b})}$$

where *n* is the ideality factor, *k* is the Boltzmann constant, *T* is the temperature, *q* is the electronic charge, $I_{s1}$ is the saturation current, and $I_{b}$ is the bias current.

The junction capacitance $C_{j}$ is defined as:

(31) $$C_{j}=\left\{\begin{array}{cc} \frac{C_{j0}}{\sqrt{1+\frac{V_{cj}}{V_{Rj}}}}+C_{d1} & \mathrm{when}\;V_{cj}>0\\ \frac{C_{j0}}{\sqrt{1+\frac{V_{cj}}{V_{Rj}}}} & \mathrm{for}\;\mathrm{the}\;\sec\mathrm{ond}\;\mathrm{case}\;\mathrm{when}\;-V_{\mathrm{on}}\leq V_{cj}\leq 0 \end{array}\right.$$

where $C_{j0}$ is the zero-bias junction capacitance, $V_{cj}$ is the junction voltage, $V_{Rj}$ is the reverse breakdown voltage, and $C_{d1}$ is the diffusion capacitance.

Schottky diodes, with metal–semiconductor junctions, offer lower threshold voltages than semiconductor–semiconductor junctions, outperforming p–n junction diodes in I–V characteristics with a 4:1 reverse bias breakdown voltage and a forward bias turn-on voltage around 0.2 V. Engineering challenges in rectifiers involve generating usable output voltage from low input power, with Schottky diodes preferred for their low-voltage application suitability and high-speed operation due to their unipolar nature, avoiding charge storage issues of bipolar diodes [76].

Schottky diodes, characterized by their lower metal–semiconductor (MS) barrier height compared to PN diodes, are the go-to choice for low-voltage applications due to their unipolar nature. This feature enables high-speed operations free from the charge storage issues seen in bipolar PN junction and PIN diodes, with capacitive loading being the primary factor affecting switching speed. The operational efficiency of Schottky diodes, determined by conduction resistance, junction capacitance, and saturation current, benefits significantly from their low turn-on voltage. However, elevated voltages can induce nonlinearity, leading to the generation of significant harmonic signals that detract from RF-to-DC conversion efficiency.

Owing to their fast-switching capabilities and minimal voltage drop in comparison to traditional p–n diodes, various microwave Schottky diode models such as HSMS-2852, HSMS-2822, HSMS-285C, HSMS-2860, and SMS7630 have been tailored to meet specific application needs across different sectors [66]. The HSMS-286C double diode, built on a Rogers4350B substrate, operates within a frequency range of 2 to 3.05 GHz. It has dimensions of 25 × 13 mm^2^ and achieves 60% efficiency at 17 dBm, with load values ranging from 620 to 2700 ohms [60].

Another notable design is the HSMS-2820 full-wave voltage doubler, built on an FR4 substrate. This device supports multiple frequencies: 0.9 GHz, 1.8 GHz, 3.5 GHz, 5.5 GHz, and 7.3 GHz, and provides efficiencies greater than 78% over a power range of −10 to 30 dBm, with a load of 5 kΩ [66].

The HSMS-286 half-wave rectifier, also using a Rogers4350 substrate, operates between 2 and 3 GHz, with dimensions of 36 × 35 mm^2^. It delivers efficiencies exceeding 40% at 10 dBm and handles power levels from 0 to 10 dBm, with a load of 400 ohms [75].

The SMS7630 voltage doubler, constructed on an RT6002 substrate, operates within the frequency ranges of 1.7–1.8 GHz and 2.1–2.7 GHz. It achieves efficiencies between 55% and 65% and has dimensions of 145 × 145 × 1.52 mm^3^, with a load of 2 kΩ [77]. For higher-frequency applications, the HSMS-2860 shunt series diode, using a RO4003C substrate, functions at 2.38 GHz and 2.45 GHz. It handles power levels from −20 to 10 dBm and achieves an efficiency of 75.3% [118]. The SMS7630-079 single-series diode, featuring a Rogers5880 substrate, operates at 1.8 GHz, with efficiencies of 21.1% at −20 dBm and 6.9% at −30 dBm, and has a load of 6 kΩ [119].

The HSMS-285C rectifier, available as both a 1-stage and 3-stage Dickson rectifier and built on an FR4 substrate, operates at 1 GHz, achieving a high efficiency of 77% with a load of 14.61 kΩ [120]. For frequencies up to 26.5 GHz, the MA4E-1319 full-wave voltage doubler, using a textile substrate, provides 12% efficiency at 10 dBm, with dimensions of 32.6 × 16 mm^2^ and a load of 630 ohms [121].

A self-tunable artificial transmission line combining the HSMS-2820 and HSMS-2850 diodes on a Taconic RF-35 substrate operates at 0.9 GHz, achieving 70% efficiency with an impedance of 390 ohms [122]. The HSMS-2860 branch two voltage doubler, employing a Rogers5880 substrate, functions at 0.866 GHz, 0.915 GHz, and 2.45 GHz, delivering efficiencies of 65%, 62%, and 60%, respectively, with a load of 10 kΩ [123].

The SMS7630 multiple diode configuration on a Rogers5880 substrate covers frequencies from 1.84 GHz to 5.8 GHz, offering efficiencies ranging from 28.3% to 65%, depending on the frequency [124]. The HSMS-285C Greinacher full-wave rectifier, with an FR4 substrate, operates at 1.85 GHz and provides 40% efficiency with a load of 4.7 kΩ [125].

The HSMS-2862-TRI diode, a voltage doubler using a RogersRO3003 substrate, functions across a broad frequency range from 0.06 GHz to 3.8 GHz, achieving an efficiency of 77.3% [126]. The combined HSMS-2862 and SMS7630 voltage doubler on an FR4 substrate operates at 0.915 GHz, 1.8 GHz, and 2.4 GHz, delivering efficiencies of 74.9%, 71.2%, and 60%, respectively, with dimensions of 24 × 8.8 cm^2^ and a load of 1500 ohms [127].

#### Summary of Schottky Rectifier Radio Frequency Energy Harvesters

Table 10 highlights several performance metrics for Schottky rectifiers used in RF energy harvesting systems. The significant findings include high power conversion efficiency (PCE) at specific frequencies and high input power levels. Schottky rectifiers have a low turn-on voltage, making them highly efficient for harvesting low-power RF signals. Additionally, some have compact sizes, making them ideal for integration into small and portable devices.

Despite these advantages, several deficiencies are evident. Schottky rectifiers are often optimized for specific frequency ranges, limiting their effectiveness across broader frequency spectrums. Their performance can degrade significantly with variations in load and input power, leading to inefficiencies in fluctuating RF environments. Parasitic series resistance and inductance can cause substantial power losses, reducing overall efficiency. Moreover, manufacturing errors can affect performance, particularly at microwave frequencies where the wavelength is close to the dimension of the microstrip line.

The identified deficiencies suggest several areas for improvement in Schottky rectifier designs. Future designs should aim to increase the operational bandwidth of Schottky rectifiers to enhance their versatility across different RF applications. Developing rectifiers that maintain high efficiency across varying input power levels and loads can help mitigate performance degradation in dynamic RF environments. Additionally, exploring advanced materials, design techniques, and models to minimize parasitic losses can enhance overall power conversion efficiency.

### 5.3. Research Challenges on Rectifier

An effective RF energy harvesting (RFEH) system should capture energy across various frequencies and power levels, deliver high output voltage with minimal power, and maintain a compact form. Current advancements in RFEH are focused on identifying research directions and addressing existing challenges [128].

The challenge lies in accurately determining parasitic and conduction losses, harmonic losses, and manufacturing errors, which can lead to low efficiency and impedance mismatches.

#### 5.3.1. Broadband Design Challenges

RFEH systems require rectifiers capable of functioning within designated frequency ranges to harvest energy from any accessible band. However, many rectifiers face challenges in efficiently covering the full spectrum. Since many frequency bands are closely spaced, a broadband rectifier is anticipated to deliver effective performance across broad or multiple bands.

#### 5.3.2. Rectifiers with Wide Power Range Challenges

The received power in wireless power transfer (WPT) systems is inversely proportional to the square of the transmitter-to-receiver distance. Thus, variations in the receiver’s position have an impact on the rectenna’s input power level. The fluctuating ambient RF power level in a WEH system affects rectifier performance. The relationship between the load resistance and the maximum power handling capability of the rectifier, or critical input power (Pc), is inverse. Changes in load have an impact on the rectifier’s capacity to handle power and may result in impedance mismatches, which lower PCE. Rectifiers must therefore minimize impedance fluctuations and sustain PCE by becoming less sensitive to input RF power and load variations.

#### 5.3.3. Improving Power Conversion Efficiency (PCE)

Enhancing PCE in RFEH systems involves reducing energy usage and losses. Innovations such as Schottky diodes and CMOS technology help minimize power consumption. Additional components like TFETs and SC-Schottky diodes further reduce energy use. Efficiency can also be improved by optimizing model performance, such as reconfigurable and distributed matching networks for operation across diverse frequency bands. Co-designing antennas and rectifiers to eliminate matching networks and developing new rectifier topologies for better PCE are crucial strategies. To achieve broader bandwidth and input power range, RFEH circuits should support a wide dynamic range. Multiband and array antennas cater to systems requiring extensive bandwidth and broad input ranges. Unique matching network topologies can address the nonlinear input impedance issues of rectifiers, expanding bandwidth and power range capabilities. Machine learning can simplify adjusting transmission line dimensions for improved matching, while reconfigurable rectifiers can adapt to various dynamic ranges and bandwidth requirements.

#### 5.3.4. Advanced Techniques and Tools

Fast and precise Maximum Power Point Tracking (MPPT) algorithms and artificial intelligence (AI) tools are essential for maintaining high PCE across a wide dynamic range, with simplified control circuits for power-aware Power Management Units (PMUs) ensuring consistent performance and system efficiency. Optimizing the electromagnetic field of the substrate through iterative simulations and integrating artificial intelligence for antenna design can maximize RFEH system performance. Co-designing antennas, matching networks, and rectifiers can enhance sensitivity and PCE while reducing system size, offering a comprehensive approach to improving RFEH systems’ efficiency and functionality.

## 6. Far-Field Rectenna: Power Harvesting and Conversion Efficiency

Rectennas are critical components in far-field radio frequency energy harvesting (RFEH) systems, converting ambient RF energy into DC power and allowing low-power devices to operate when conventional power sources are unavailable or unfeasible [8]. While previous parts addressed the design and functionality of antennas and rectifiers in RFEH systems, this section focuses on the power harvested by rectennas and the issues associated with optimizing power usage [19]. Far-field rectennas must efficiently capture energy delivered over long distances, and their performance is vital in applications such as distant sensors, medical implants, and satellite communications, where reducing power loss is essential for prolonged operation [129].

Table 11 outlines the power consumption of common electronic devices and sensors, ranging from GPS receiver chips at 15 mW to RF transmission at sub-μW levels. In far-field RF energy harvesting (RFEH) systems, rectennas must supply adequate power to support these devices. High-demand components like GPS chips and cell phones in standby mode present a challenge for RF harvesting [130], while lower-power sensors like accelerometers, pressure, humidity, and temperature sensors, which require between 0.32 mW and 27 μW, are more suitable for energy harvesting applications. This demonstrates the potential of RFEH systems to power low-energy devices, particularly in IoT and sensor networks.

Multiband rectennas [64] are designed for certain frequencies, such as the ISM bands at 915 MHz and 2.45 GHz, and are extremely efficient within these regions. Broadband rectennas, on the other hand, capture energy across a broader frequency range. Rectennas use power combiners such as DC, RF, and hybrid to improve energy harvesting. DC combiners combine the rectified DC output, whereas RF combiners blend RF signals before rectification, decreasing energy loss. Hybrid combiners combine both technologies, allowing for harvesting at several frequencies and power levels. This paper [64] presents a triple-band differential rectenna designed for RF energy harvesting. It operates in the 2.1 GHz (UMTS), 2.4–2.48 GHz (WLAN/Wi-Fi), and 3.3–3.8 GHz (WiMAX) frequency bands. The rectenna consists of a differentially fed multiband slot antenna with peak gains of 7 dBi at 2 GHz, 5.5 dBi at 2.5 GHz, and 9.2 dBi at 3.5 GHz. A triple-band rectifier using a Villard voltage doubler and interdigital capacitors is implemented, achieving a peak RF-to-DC conversion efficiency of 68%. When integrated, the rectenna shows maximum conversion efficiencies of 53% at 2 GHz, 31% at 2.5 GHz, and 15.56% at 3.5 GHz. Conventional antennas [131] are constrained by narrow beamwidths, but high-gain antennas improve power collection. To reduce the requirement for sophisticated beamforming networks, we present a traveling-wave grid-array antenna (GAA) with two isolated ports and symmetrically inclined beams. A prototype was created and tested, displaying increased sensitivity and wide-angle coverage. With a power density of 1 µW/cm^2^, the dual-beam system produced more than 100 µW of DC power, expanding the harvesting range beyond 70°. At 2.45 GHz, the rectenna produced a DC output of 3.6–203.8 µW and achieved a maximum RF-to-DC conversion efficiency of 16.3–45.3%. Power densities ranged from 0.052 to 1 µW/cm^2^. This article [132] introduces a six-beam antenna design that removes the need for a complex feeding system. Integrating a surface waveguide layer and a triangular patch array, it achieves a peak gain of 8.3 dBi and 3 dB beamwidths of 62° in the H-plane and 56° in the E-plane, providing full azimuthal coverage. The energy harvester effectively gathers electromagnetic energy from various horizontal directions and substantially boosts DC power output in the presence of multiple EM transmitters.

The receiving array is optimized for power transmission using quadratic programming, offering adjustable angular coverage. The SMS 7630 diode improves rectification at low incident power. A six-element patch array and 5.8 GHz rectifier were tested, showing stable DC output as the energy source moves between $-45^{\circ }$ and $+45^{\circ }$ in the H-plane [133]. The hybrid power combining array has a beam-forming matrix and a DC power management network (PMN), and its normalized DC output is compared to traditional approaches using incident wave angles and received DC power. Four patch antennas were designed using a 4 × 4 Butler matrix with quadrature hybrids. A reconfigurable voltage doubler rectifier and DC PMN convert RF energy into DC and provide the necessary voltage to the load [134]. Table 12 summarizes various rectenna designs, highlighting their performance metrics and the types of combiners used, illustrating the efficiency and power generated by the rectennas.

## 7. Conclusions

In conclusion, this review emphasizes the critical role of rectenna technology in advancing IoT and 5G systems, which demand sustainable and low-maintenance solutions. RF energy harvesting is pivotal for enabling real-time data acquisition and contributing to a carbon-free future.

The key challenges are as follows:

Rectifier Performance: Existing rectifiers are typically optimized for narrow frequency bands, leading to inefficiencies when handling a broad range of frequencies. Performance can also degrade with variations in input power and load.

Antenna Miniaturization: Although miniaturization is essential for compact devices, it often compromises performance and efficiency. Achieving effective circular polarization in reduced form factors presents additional challenges.

Impedance Matching: Efficient impedance matching across varying frequencies and power levels remains difficult, impacting overall system effectiveness.

Our recommendations are as follows:

Develop Versatile Rectifiers: Design rectifiers that perform efficiently across a wide frequency range and are less sensitive to variations in input power and load. Explore innovations such as adaptive topologies and advanced materials.

Advance Miniaturization Techniques: Research should focus on enhancing miniaturization while maintaining performance, including developing new materials and designs that improve efficiency.

Optimize Impedance Matching: Create advanced impedance matching networks capable of dynamically adjusting to changes in frequency and power levels. Implementing machine learning algorithms for real-time optimization could be advantageous.

Explore Innovative Materials: Investigate new materials, such as advanced metamaterials and emerging semiconductor technologies, to enhance the performance of rectifiers and antennas.

Integrate Machine Learning: Utilize machine learning techniques to optimize design parameters, such as antenna dimensions and rectifier topologies, to improve adaptability and performance under various operating conditions.

Addressing these challenges and implementing these recommendations will be crucial for advancing RF energy harvesting technologies, enhancing the efficiency and sustainability of IoT, 5G systems, and Industrial 5.0 in the future.

## Figures and Tables

**Table 1:** Characteristics and performance of different types of energy harvesting (EH) technologies.

Type of EH	Solar [15]	Thermal [16]	Wind [17]	Piezoelectric [18]	RF [19]
**Power Source**	Sun	Solar, equipment malfunctions, and physical attrition	Airspeed	Fluctuations in strength and oscillations	Broadcasting channels, cellular networks, and infrastructure
**Access/Availability**	Sunlight hours (4–8)	Ongoing system operations	Weather-dependent	Based on activity	Daily
**Scavenging process**	Photovoltaic/solar cell	Temperature, photoelectric effect	Motor drive, wind turbine	Piezoelectric devices with power electronics	Rectenna
**Avg. Power density**	100 μW/cm^2^	60 μW/cm^2^	177 μW/cm^2^	250 μW/cm^2^	40 μW/cm^2^
**Features**	Hard to manage	Unmanageable, hard to predict	Unmanageable, unpredictable	Manageable	Somewhat manageable
**Efficiency**	11.7–26.7%	5–15%	-	5–30%	0.4–50%
**Uses**	External IoT devices, hubs	Body sensors	IoT sensors, base stations	Wearables, sensors	RFID, wearables
**Merits**	Abundant energy, tech progress	Small-scale harvesters	Energy from light wind	High efficiency, stable voltage output	Compact, available circuits
**Demerits**	Weather-dependent, large area needed	Thermal compatibility, high power need	Availability varies, bulky systems	Expensive, material-dependent	Interference affects power, signal loss

**Table 2:** Types of wireless power transfer.

Types of Wireless Power Transfer	Field Region	Design Method	Effective Distance	Efficiency	Uses	Merits	Di-Merits
**RF Harvesting [8]**	Far-Field	Antennas	Depends on frequency, works over meters to kilometers.	1% to 85%	Low-power devices, body wireless sensors, wearables	Low radiative effect due to small RF density	High RF can be hazardous, low efficiency
**Inductive Coupling [33]**	Near-field, non-radiative	Coils	Ranges from mm to cm	10–60% at 15–500 KHz	Mobiles, cards, recharge stations	Easy to implement and safe	Limited range, heating, alignment issues
**Magnetic Resonance Coupling [34]**	Near-Field	Resonators	Several millimeters to several meters	20% to 90% at 50 cm to 3 m	Wireless charging, electric cars, mid-range applications	Charges multiple devices efficiently, even with misalignment	Limited range and complexity hinder mobile use

**Table 3:** Various multiband and broadband antennas for rectenna application.

Antenna	Methodology/Substrate	Frequency	Size	Gain (dBi)	RF-DC Efficiency	Ref.
Slot	Star antenna on F4B	2.45 GHz 5.8 GHz	133 × 93 mm^2^	1.48 dBi@2.4 GHz 3.83 dBi@5.8 GHz	63%@2.4 GHz 54.8%@5.8 GHz	[61]
Monopole	Corrugated microstrip on 0.508 mm R04350, $\epsilon_{r}$ = 3.48	0.9 GHz 2.1 GHz 2.36 GHz	70 × 66 mm^2^	1 dBi@0.9 GHz 2.64 dBi@2.1 GHz −0.19 dBi@2.36 GHz	42.2%@0.9 GHz 72.6%@2.1 GHz 32.8%@2.36 GHz	[62]
Meandered	Spiral antenna on FR4	(2.45–2.5 GHz)	32.8 × 9.7 × 0.8 mm³	2.83 dBi@2.45 GHz	50%	[63]
Circular	Layered circular slot on 1.6 mm FR4, $\epsilon_{r}$ = 4.4	0.908–0.922 GHz 2.35–2.50 GHz	120 × 120 mm^2^	5.41 dBi@918MHz 7.9 dBi@2.48 GHz	19%@0.9 GHz 17%@2.45 GHz	[67]
Slot	Slot antenna on copper, 1.6 mm FR4, $\epsilon_{r}$ = 4.4	2.1 GHz 2.4–2.48 GHz 3.3–3.8 GHz	120 × 120 × 30 mm³	7 dBi@2 GHz 5.5 dBi@2.5 GHz 9.2 dBi@3.5 GHz	85%@2 GHz 75%@2.49 GHz 72%@3.4 GHz	[64]
Monopole	On 1.6 mm FR4, $\epsilon_{r}$ = 4.4	0.9 GHz 1.8 GHz 2.1 GHz 2.45 GHz	130 × 80 mm^2^	2.6 dBi@0.9 GHz 3.6 dBi@1.8 GHz 3.8 dBi@2.1 GHz 4.7 dBi@2.45 GHz	25%@1.8 GHz 27%@2.15 GHz	[65]
Fed square patch	Fractal patch on 1.6 mm FR4	0.8–1.2 GHz 1.6–2.1 GHz 2.2–2.8 GHz 3.1–4.0 GHz 5.3–6.4 GHz 7.0–7.8 GHz	60 × 60 × 1.6 mm³	1 dBi@900MHz 3 dBi@2 GHz 5 dBi@5.5 GHz 4 dBi@7 GHz	28%@900MHz 24%@2.5 GHz 9%@1.8 GHz 17%@3.5 GHz 13%@5.5 GHz 36%@7.5 GHz	[66]

**Table 4:** Antenna arrays for rectenna applications.

AntennaArray	Methodology/Substrate	Frequency	Size	Gain (dB)	RF-DC Efficiency	Ref.
Quasi-Yagi	Quasi-Yagi on 0.762 mm Rogers 5880, $\varepsilon_{r}=2.2$	2.3–2.63 GHz	26 × 190.5 mm^2^	8.7 dBi	25%@2.45 GHz	[55]
Stacked Patch	Short pin omni on 1.6 mm FR4, $\varepsilon_{r}=4.4$	3.3–3.9 GHz	25.7 × 25.7 mm^2^	7.8 dBi@3.5 GHz	-	[68]
MIMO Array	Rectangular patch array on 1.6 mm FR4	2.32 GHz 2.8 GHz	100 × 190 mm^2^	−10.13 dBi	-	[70]
Dipole	Rogers 4350, 0.8 mm, $\varepsilon_{r}=3.48$	0.76–0.88 GHz 1.9–2.7 GHz 3.3–3.9 GHz	86 × 125 mm^2^	6.9 dBi@0.76 GHz 1.3 dBi@2.3 GHz 10.6 dBi@3.6 GHz	57% 49.5% 60.44%	[72]
Reflector	Ferrite-loaded, 1.0 mm, $\varepsilon_{r}=15$, μ=1000	1.7–2.7 GHz	380 × 350 mm^2^	8.9 dBi	-	[73]
Four-Patch Array	Unidirectional on 0.8 mm Rogers 4350, $\varepsilon_{r}=3.48$	2.45 GHz	160 × 160 × 7 mm³	12.7 dBi	81.5%	[74]
Two-element Array	Unidirectional array on FR4	(0.69–0.96) GHz Lower band (1.7–2.7 GHz) Upper band	43 × 43 mm^2^	14.65 dBi	35% Lower band 45% Upper band	[75]
Four Square Patches	Omni antenna on FR4	1.65–2.76 GHz	135 × 135 mm^2^	8.5 dBi	22%@2.45 GHz	[76]
Twelve-element Vivaldi Array	Omni antenna on 0.5 mm RT6002, $\varepsilon_{r}=6.15$	1.7–1.8 GHz 2.1–2.7 GHz	145 × 145 × 1.52 mm³	4.33 dBi@1.8 GHz 4.22 dBi@2.15 GHz 3.88 dBi@2.45 GHz	>60%	[77]

**Table 5:** Metamaterial-inspired miniaturization methods.

Methodology/Substrate	Frequency	S_11_ Bandwidth (%)	Maximum Efficiency (%)	Gain (dB)	Size	Ref.
CRLH materials on 1.0 mm RT5800, $\epsilon_{r}$ = 2.2.	2.45 GHz 5.8 GHz	-	65	1.5 dBi@2.4 GHz 5.2 dBi@5.8 GHz	30 mm radius	[84]
MSRR on 0.762 mm Rogers 5870, $\epsilon_{r}$ = 2.33.	1.7–2.67 GHz	44.1	45	8.51 dBi	0.55$\lambda_{0}$ × 0.36$\lambda_{0}$	[85]
CSRR on 1.0 mm RT5800, $\epsilon_{r}$ = 2.2.	1.8 GHz	16	-	3.1 dBi	63.8 × 83.8 mm^2^	[86]
Capacitive metasurface on 0.8 mm Rogers 4003C, $\epsilon_{r}$ = 3.55.	3.02–3.63 GHz	8.5	-	6.57 dBi	0.58$\lambda_{0}$ × 0.58$\lambda_{0}$ × 0.043$\lambda_{0}$	[87]
SRRs on 1.0 mm Rogers 3010, $\epsilon_{r}$ = 10.2.	3.02–3.63 GHz	8.5	-	6.57 dBi	4.3$\lambda_{0}$ × 4.3$\lambda_{0}$	[88]

**Table 6:** Antenna gain and bandwidth enhancement methods.

Methodology/Substrate	Frequency	S_11_ Bandwidth (%)	Maximum Efficiency (%)	Gain (dBi)	Size	Ref.
ZOR on 0.81 mm RO4003C, $\epsilon_{r}$ = 3.55	348–772 MHz	78	NA	9.2 dBi	0.46$\lambda_{0}\;\times$ 0.46$\lambda_{0}$	[90]
SRRs on 1.6 mm FR4, $\epsilon_{r}$ = 4.4	0.865–1.06 GHz 2.240–2.52 GHz 3.25–4.31 GHz 4.9–6.5 GHz	28	NA	6.74 dBi	78.6 × 42.5 mm^2^	[91]
Metamaterial on 0.635 mm Rogers 6010, $\epsilon_{r}$ = 6.15	0.915 GHz 2.45 GHz	17.8% 35.8%	NA	17.8 dBi@0.915 GHz 9.81 dBi@2.45 GHz	7 × 6 × 0.254 mm³	[92]
Near-zero-index on 1.6 mm FR4, $\epsilon_{r}$ = 4.6	0.534 GHz	2.11%	74.1%	7.27 dBi	170 × 170 mm^2^	[93]

**Table 7:** Circular polarization antenna for rectenna.

Methodology/Substrate	Frequency	S_11_ Bandwidth (%)	Maximum Efficiency (%)	Gain (dB)	Size	Ref.
Metamaterial cavity on 0.635mm RT6010, 0.762 mm Rogers 5880, $\epsilon_{r}$ = 6.15/2.2.	9.7–10.27 GHz	6.0	74.1	14.1 dBi	NA	[75]
EBG on 1.0 mm F4B, $\epsilon_{r}$ = 3.5.	12.5 GHz 14.2 GHz	6.0 4.3	47.7	23.1 dBi 24.4 dBi	150 × 150 mm^2^	[79]
Agile resonator with parasitics on 0.762 mm Rogers 5880, $\epsilon_{r}$ = 2.2.	1.39 GHz	3.92	NA	5.92 dBi	NA	[94]

**Table 8:** Isolation and mutual coupling method to improve the multi-port rectenna.

Methodology/Substrate	Frequency	S_11_ Bandwidth (%)	Maximum Efficiency (%)	Gain (dB)	In Band Isolation (dB)	Peak Isolation (dB)	Ref.
Metastrip on 0.762 mm Rogers 5880, $\epsilon_{r}$ = 2.2.	28 GHz	20.12	NA	8 dBi	22	24	[60]
EBG/DGS on 0.76 mm TLY-5 ($\epsilon_{r}$ = 2.2) and 1.6 mm FR4 ($\epsilon_{r}$ = 4.4).	28 GHz	47.7	81.9%	9 dBi	32.7	71.9	[95]
CSRR on 0.762 mm Rogers 5880, $\epsilon_{r}$ = 2.2.	25 GHz	31.8	NA	NA	32	55	[96]
CSRR with slot on 0.635 mm Rogers 6010, $\epsilon_{r}$ = 6.15.	3.6 GHz	27	3.59	NA	35	52	[97]

**Table 9:** Overview of CMOS rectifier designs for wireless energy scavenging.

Design	Frequency	Efficiency (%)	Dynamic Range (dB)	Area (mm^2^)	Ref.
CMOS Differential Rectifier	2.4 GHz	46%	25.5	1.5 × 0.47	[90]
CMOS Villard Multiplier Rectifier	400 MHz/2.4 GHz	75%	NA	NA	[106]
CMOS Passive Rectifier	0.2 GHz	70.3%	−4 to 7	0.88	[110]
CMOS Bootstrap Rectifier	434 MHz	71%	−6 to 6	0.30	[111]
Cross-Coupled CMOS Rectifier	2.45 GHz	73% (−6 dBm) 70.4% (−5.5 dBm)	−5 to 7	0.90	[113]
CMOS-CCDD Rectifier	0.9 GHz	83.7% (−18.4 dBm) 80.3% (−17 dBm)	−21 to 13	NA	[114]
Double-Sided CMOS Rectifier	0.9 GHz	66%	NA	0.088	[115]
CMOS Reconfigurable Rectifier	0.9 GHz	25%	NA	NA	[116]
CMOS Rectifier	6.78 MHz	92.2%	NA	8.0	[117]

**Table 10:** Schottky diode rectifier performance.

Diodes	Methodology/Substrate	Frequency	Size	Input Power (dBm)	RF-DC Efficiency (%)	Load (Ω)	Ref.
HSMS 286C	Double diode on Rogers 4350B	2–3.05 GHz	25 × 13 mm^2^	0 to 25	60%@17 dBm	620 to 2700	[60]
HSMS 2820	Full-wave doubler on FR4	0.9 GHz, 1.8 GHz, 3.5 GHz, 5.5 GHz, 7.3 GHz	-	−10 to 30	>78%	5 K	[66]
HSMS 286	Half-wave rectifier on Rogers 4350	2–3 GHz	36 × 35 mm^2^	0–10	>40%@10 dBm	400	[75]
SMS 7630	Voltage doubler on RT6002	1.7–1.8 GHz, 2.1–2.7 GHz	145 × 145 × 1.52 mm³	3	55–65%	2 K	[77]
HSMS 2860	Shunt diode on RO4003C	2.38 GHz, 2.45 GHz	-	−20 to 10	75.3%@dBm	4.47 K	[118]
SMS 7630-079	Single diode on Rogers 5880	1.8 GHz	32 × 32 mm^2^	−40 to 20	21.1%@−20 dBm, 6.9%@−30 dBm	6 K	[119]
HSMS 285C	1-stage/3-stage Dickson rectifier on FR4	1 GHz	-	20	77%	14.61 K	[120]
MA4E-1319	Full-wave doubler on textile	20–26.5 GHz	32.6 × 16 mm^2^	10	12%	630	[121]
HSMS 2820 and HSMS-2850	Self-tunable line on Taconic RF-35	0.9 GHz	0.195 × 0.073 $\lambda^{2}$	13	70%	390	[122]
HSMS 2860	Branch doubler on Rogers 5880	0.866 GHz, 0.915 GHz, 2.45 GHz	3.5 × 2.6 cm^2^	−30 to 0	65%@0.866 GHz, 62%@0.915 GHz, 60%@2.45 GHz	10 K	[123]
SMS 7630	Multiple diodes on Rogers 5880	1.84 GHz 2.04 GHz 2.36 GHz 2.54 GHz 3.3 GHz 4.76 GHz 5.8 GHz	54 × 42 mm^2^	−20 to 4	65%@4 dBm, 28.3–65% across frequencies	1300	[124]
HSMS 285C	Greinacher rectifier on FR4	1.85 GHz	70 × 70 × 1.6 mm³	20	40%	4.7 K	[125]
HSMS- 2862-TRI	Voltage doubler with virtual battery on Rogers RO3003	0.06–3.8 GHz	20 × 7.4 mm^2^	23	77.3%	1.3 K	[126]
HSMS 2862 SMS 7630	Voltage doubler with dual-band transmission on FR4	0.915 GHz 1.8 GHz 2.4 GHz	24 × 8.8 cm^2^	17	74.9%, 71.2%, 2.45 GHz	1500	[127]

**Table 11:** Average power consumption of typical electronic devices and sensors [130].

Device	Power Consumption (mW)
GPS Module	15
Mobile Device (Sleep Mode)	8.1
Optical Heart Rate Sensor	1.47
Air Moisture Sensor	1
Pressure Sensing Device	0.5
3D Accelerometer	0.32
Temperature Sensor	0.027
A/D Conversion	0.001
RF Transmission	Sub-μW

**Table 12:** Performance of far-field rectenna system and power combining method.

Rectenna Design	Frequency	Load	Size	Gain (dBi)	Rectifier	RF-DC Efficiency (%) Power	Ref.
Multiband Differentially fed	2 GHz 2.5 GHz 3.5 GHz	2–1.5kΩ	$2.94\times 2.94\times 0.027\;\lambda^{3}$	7@2GHz 5.5@2.5GHz 9.2@3.5GHz	Villard Voltage Doubler	53%@2GHz for $P_{\mathrm{in}}=-13\;\mathrm{dBm}$, 30.7% at 2.5GHz for $P_{\mathrm{in}}=12\;\mathrm{dBm}$, max power is 26.58 μW.	[64]
Grid-Array DC combiner	2.45 GHz	6kΩ	$3.87\times 5.77\times 0.274\;\lambda^{3}$	15.5	Voltage Doubler	58%@2.45GHz for $P_{\mathrm{in}}=-13.2\;\mathrm{dBm}$, max power is 83.3 μW.	[131]
Patch Array DC combiner	5.2 GHz	1kΩ	$2.39\times 2.06\times 0.073\;\lambda^{3}$	8.3	Single series	57.7%@5.2GHz for $P_{\mathrm{in}}=7\;\mathrm{dBm}$, max power is 0.69mW.	[132]
Square Patch RF combiner	5.8 GHz	1.18kΩ	$2.76\times 0.76\times 0.02\;\lambda^{3}$	6.5	Single shunts	60.1%@5.8GHz for $P_{\mathrm{in}}=0\;\mathrm{dBm}$, max power is 382 μW.	[133]
Suspended Patch Hybrid combiner	2.45 GHz	6.2kΩ	$1.7\times 4.11\times 0.085\;\lambda^{3}$	5.9	Voltage Doubler	55.3%@2.45GHz for $P_{\mathrm{in}}=-4\;\mathrm{dBm}$, max power is 250 μW.	[134]

## List of Acronyms

**Acronyms**	**Full Form**
RF, WEH	Radio Frequency, Wireless Energy Harvesting
RFEH, WPT	RF Energy Harvesting, Wireless Power Transfer
PCE, DC	Power Conversion Efficiency, Direct Current
CMOS	Complementary Metal-Oxide-Semiconductor
AC, Qi	Alternating Current, Charging Flow
EM, RFID	Electromagnetic, RF Identification
GSM, UMTS	Global System for Mobile, Universal Mobile Telecommunications System
LoRaWAN	Long Range Wide Area Network
TSOP, WLAN	Tapered Slot Patch, Wireless Local Area Network
LTE, WiMAX	Long-Term Evolution, Wireless Microwave Access
FR4, MIMO	Flame-Retardant Material, Multiple Input Multiple Output
ISM, TD-LTE	Industrial, Scientific, Medical, Time Division LTE
DNG, MNG	Double-Negative, Magnetic-Negative
SNG, EPG	Single-Negative, Epsilon-Negative
CRLH-TL, RH-TL	Composite Right/Left-Hand Transmission Line, Right-Handed
FSS, CSRR	Frequency Selective Surface, Complementary Split-Ring Resonator
IMN, CMOS-CCDD	Impedance Matching Network, CMOS Capacitive Coupled Device

## References

[1] Karthick Raghunath K., Koti M., Sivakami R., Vinoth Kumar V., NagaJyothi G., Muthukumaran V. (2022). Utilization of IoT-assisted computational strategies in wireless sensor networks for smart infrastructure management. Int. J. Syst. Assur. Eng. Manag..

[2] Khan S., Lee W.-K., Hwang S.O. (2022). AEchain: A Lightweight Blockchain for IoT Applications. IEEE Consum. Electron. Mag..

[3] Dash D. (2022). Geometric Algorithm for Finding Time-Sensitive Data Gathering Path in Energy Harvesting Sensor Networks. IEEE Trans. Intell. Transp. Syst..

[4] Kim S., Kim B., Shah B., Ullah S., Kim K.I. (2021). Survey on communication for mobile sinks in wireless sensor networks: Mobility pattern perspective. J. Internet Technol..

[5] Bhaskarwar R.V., Pete D. (2021). Cross-Layer Design Approaches in Underwater Wireless Sensor Networks: A Survey. SN Comput. Sci..

[6] Gupta S., Jana P. (2015). Energy Efficient Clustering and Routing Algorithms for Wireless Sensor Networks: GA Based Approach. Wirel. Pers. Commun..

[7] Rajagopalan R., Varshney P. (2006). Data-aggregation techniques in sensor networks: A survey. IEEE Commun. Surv. Tutor..

[8] Chen D., Li R., Xu J., Li D., Fei C., Yang Y. (2023). Recent progress and development of radio frequency energy harvesting devices and circuits. Nano Energy.

[9] Sharma P., Singh A. (2023). A survey on RF energy harvesting techniques for lifetime enhancement of wireless sensor networks. Sustain. Comput. Inform. Syst..

[10] Alibakhshikenari M., Virdee B., Azpilicueta L., Naser-Moghadasi M., Akinsolu M., See C., Liu B., Abd-Alhameed R.A., Falcone F. (2020). A Comprehensive Survey of ‘Metamaterial Transmission-Line Based Antennas: Design, Challenges, and Applications’. IEEE Access.

[11] Esmail B., Koziel S., Szczepanski S. (2022). Overview of Planar Antenna Loading Metamaterials for Gain Performance Enhancement: The Two Decades of Progress. IEEE Access.

[12] Hussain M., Awan W., Alzaidi M., Hussain N., Ali E., Falcone F. (2023). Metamaterials and Their Application in the Performance Enhancement of Reconfigurable Antennas: A Review. Micromachines.

[13] Chun A., Ramiah H., Mekhilef S. (2022). Wide Power Dynamic Range CMOS RF-DC Rectifier for RF Energy Harvesting System: A Review. IEEE Access.

[14] Yildiz F. (2009). Potential Ambient Energy-Harvesting Sources and Techniques. J. Technol. Stud..

[15] Green M., Dunlop E., Hohl-Ebinger J., Yoshita M., Kopidakis N., Hao X. (2020). Solar cell efficiency tables (version 56). Prog. Photovoltaics Res. Appl..

[16] Stevens J. (1999). Optimized Thermal Design of Small *Δ*T Thermoelectric Generators.

[17] Mitcheson P., Green T., Yeatman E., Holmes A. (2004). Architectures for Vibration-Driven Micropower Generators. J. Microelectromech. Syst..

[18] Roundy S., Wright P., Pister K. Micro-Electrostatic Vibration-to-Electricity Converters. Proceedings of the Microelectromechanical Systems, ASME 2002 International Mechanical Engineering Congress and Exposition.

[19] Piñuela M., Mitcheson P., Lucyszyn S. (2013). Ambient RF energy harvesting in urban and semi-urban environments. IEEE Trans. Microw. Theory Tech..

[20] Srivastava V., Sharma A. (2024). A Coil Rectenna Array Design to Harvest All H-Field Components for Lateral Misalignment Tolerant Wireless Powering of Bio-Medical Implant Devices. IEEE J. Electromagn. RF Microwaves Med. Biol..

[21] Kumar M., Kumar S., Jain S., Sharma A. (2023). A Plug-in Type Integrated Rectenna Cell for Scalable RF Battery Using Wireless Energy Harvesting System. IEEE Microw. Wirel. Technol. Lett..

[22] Samal P., Chen S., Fumeaux C. (2023). Wearable Textile Multiband Antenna for WBAN Applications. IEEE Trans. Antennas Propag..

[23] Kiourti A., Nikita K. (2012). A review of implantable patch antennas for biomedical telemetry: Challenges and solutions. IEEE Antennas Propag. Mag..

[24] Zada M., Shah I., Nasir J., Basir A., Yoo H. (2023). Empowering Remote Patient Monitoring with a Dual-Band Implantable Rectenna System for Wireless Power and Data Transfer. IEEE Trans. Antennas Propag..

[25] Dibner B. (2013). Oersted and the discovery of electromagnetism. Electr. Eng..

[26] Levin M., Miller M. (1981). Maxwell’s ‘Treatise On Electricity and Magnetism’. Sov. Phys.-Uspekhi.

[27] Janik A. (2000). Heinrich hertz: Classical Physicist, Modern Philosopher. Davis Baird, R. I. G. Hughes, Alfred Nordmann. Isis.

[28] Carlson W., Wenaas E. (2014). Tesla: Inventor of the electrical age. IEEE Technol. Soc. Mag..

[29] Shinohara N. (2021). History and Innovation of Wireless Power Transfer via Microwaves. IEEE J. Microw..

[30] Surender D., Khan T., Talukdar F., Antar Y. (2022). Rectenna Design and Development Strategies for Wireless Applications: A Review. IEEE Antennas Propag. Mag..

[31] Zhou X., Zhao J., Liang N., Wu S., Wang J. (2016). Analysis of WiTricity corporation’s wireless charging patents. Gaojishu Tongxin/Chinese High Technol. Lett..

[32] Theodoridis M. (2012). Effective capacitive power transfer. IEEE Trans. Power Electron..

[33] Sanislav T., Mois G., Zeadally S., Folea S. (2021). Energy Harvesting Techniques for Internet of Things (IoT). IEEE Access.

[34] Asiful Huda S., Arafat M., Moh S. (2022). Wireless Power Transfer in Wirelessly Powered Sensor Networks: A Review of Recent Progress. Sensors.

[35] Cho Y., Lee S., Kim D.H., Kim H., Song C., Kong S., Park J., Seo C., Kim J. (2018). Thin Hybrid Metamaterial Slab with Negative and Zero Permeability for High Efficiency and Low Electromagnetic Field in Wireless Power Transfer Systems. IEEE Trans. Electromagn. Compat..

[36] (2021). FCC Rules for Unlicensed Wireless Equipment Operating in the ISM Bands. https://Afar.Net/Tutorials/Fcc-Rules/.

[37] Soyata T., Copeland L., Heinzelman W. (2016). RF Energy Harvesting for Embedded Systems: A Survey of Tradeoffs and Methodology. IEEE Circuits Syst. Mag..

[38] Liu C., Zhang Y., Liu X. (2018). Circularly Polarized Implantable Antenna for 915 MHz ISM-Band Far-Field Wireless Power Transmission. IEEE Antennas Wirel. Propag. Lett..

[39] Kim J., Cho S., Kim H.J., Choi J.W., Jang J., Choi J. (2016). Exploiting the Mutual Coupling Effect on Dipole Antennas for RF Energy Harvesting. IEEE Antennas Wirel. Propag. Lett..

[40] Zeng M., Andrenko A., Liu X., Li Z., Tan H. (2017). A Compact Fractal Loop Rectenna for RF Energy Harvesting. IEEE Antennas Wirel. Propag. Lett..

[41] Kamoda H., Kitazawa S., Kukutsu N., Kobayashi K. (2015). Loop antenna over artificial magnetic conductor surface and its application to dual-band RF energy harvesting. IEEE Trans. Antennas Propag..

[42] Schaubert D., Kasturi S., Boryssenko A., Elsallal W. Vivaldi antenna arrays for wide bandwidth and electronic scanning. Proceedings of the IET Seminar Digest.

[43] Yadav H., Ray K., Gupta M. (2021). Differential multiresonator stacked microstrip antenna for wireless energy harvesting. Int. J. RF Microw. Comput.-Aided Eng..

[44] Anandkumar D., Sangeetha R. (2020). Design and analysis of aperture coupled micro strip patch antenna for radar applications. Int. J. Intell. Netw..

[45] Amer A., Sapuan S., Nasimuddin N., Alphones A., Zinal N. (2020). A Comprehensive Review of Metasurface Structures Suitable for RF Energy Harvesting. IEEE Access.

[46] Shen S., Zhang Y., Chiu C.Y., Murch R. (2019). An Ambient RF Energy Harvesting System Where the Number of Antenna Ports Is Dependent on Frequency. IEEE Trans. Microw. Theory Tech..

[47] Balanis C. (2016). Antenna Theory: Analysis and Design.

[48] Mugitani A., Sakai N., Hirono A., Noguchi K., Itoh K. (2022). Harmonic Reaction Inductive Folded Dipole Antenna for Direct Connection with Rectifier Diodes. IEEE Access.

[49] Boursianis A., Papadopoulou M., Pierezan J., Mariani V., Coelho L., Sarigiannidis P., Koulouridis S., Goudos S.K. (2021). Multiband Patch Antenna Design Using Nature-Inspired Optimization Method. IEEE Open J. Antennas Propag..

[50] Liu P., Jiang W., Hu W., Sun S., Gong S. (2021). Wideband Multimode Filtering Circular Patch Antenna. IEEE Trans. Antennas Propag..

[51] Biswas B., Ghatak R., Poddar D. (2015). UWB monopole antenna with multiple fractal slots for band-notch characteristic and integrated Bluetooth functionality. J. Electromagn. Waves Appl..

[52] Midya M., Bhattacharjee S., Mitra M. (2019). Broadband Circularly Polarized Planar Monopole Antenna with G-Shaped Parasitic Strip. IEEE Antennas Wirel. Propag. Lett..

[53] Sabhan D., Nesamoni V., Thangappan J. (2021). An Efficient 2.45 GHz Spiral Rectenna without a Matching Circuit for RF Energy Harvesting. Wirel. Pers. Commun..

[54] Song M., Zhang X., Wang Z., Xu D., Loh T., Gao S. (2024). 3D-printed Spiral Inverted Ridge Circularly Polarized Horn Antenna with One Octave Bandwidth. IEEE Antennas Wirel. Propag. Lett..

[55] Hu Y., Sun S., Xu H. (2020). Compact Collinear Quasi-Yagi Antenna Array for Wireless Energy Harvesting. IEEE Access.

[56] Soboll P., Wienstroer V., Kronberger R. (2015). Stacked Yagi-Uda Array for 2.45-GHz Wireless Energy Harvesting. IEEE Microw. Mag..

[57] Nasimuddin, Esselle K., Verma A. (2008). Wideband high-gain circularly polarized stacked microstrip antennas with an optimized C-type feed and a short horn. IEEE Trans. Antennas Propag..

[58] Pereira E., Paz H., Silva V., Cambero E., Casella I., Capovilla C. (2021). An Antipodal Vivaldi Antenna with Elliptical Slots for Low-Cost Rectenna Applications. J. Circuits Syst. Comput..

[59] Shi Y., Fan Y., Li Y., Yang L., Wang M. (2019). An efficient broadband slotted rectenna for wireless power transfer at LTE band. IEEE Trans. Antennas Propag..

[60] Zhang Y., Deng J., Li M., Sun D., Guo L. (2019). A MIMO Dielectric Resonator Antenna with Improved Isolation for 5G mm-Wave Applications. IEEE Antennas Wirel. Propag. Lett..

[61] Bhatt K., Kumar S., Kumar P., Tripathi C. (2019). Highly Efficient 2.4 and 5.8 GHz Dual-Band Rectenna for Energy Harvesting Applications. IEEE Antennas Wirel. Propag. Lett..

[62] Yang L., Zhou Y., Zhang C., Yang X., Yang X.X., Tan C. (2018). Compact Multiband Wireless Energy Harvesting Based Battery-Free Body Area Networks Sensor for Mobile Healthcare. IEEE J. Electromagn. RF Microwaves Med. Biol..

[63] Yaseen R., Naji D., Shakir A. (2021). Optimization Design Methodology of Broadband or Multiband Antenna for RF Energy Harvesting Applications. Prog. Electromagn. Res. B.

[64] Chandravanshi S., Sarma S.S., Akhtar M. (2018). Design of Triple Band Differential Rectenna for RF Energy Harvesting. IEEE Trans. Antennas Propag..

[65] Khemar A., Kacha A., Takhedmit H., Abib G. (2018). Design and experiments of a dual-band rectenna for ambient RF energy harvesting in urban environments. IET Microwaves Antennas Propag..

[66] Singh N., Kanaujia B., Beg M., Mainuddin, Kumar S., Choi H., Kim K. (2019). Low profile multiband rectenna for efficient energy harvesting at microwave frequencies. Int. J. Electron..

[67] Jie A., Nasimuddin N., Karim M., Chandrasekaran K. (2019). A dual-band efficient circularly polarized rectenna for RF energy harvesting systems. Int. J. RF Microw. Comput.-Aided Eng..

[68] Fang Y., Wu L., Qiu L., Zhang Y. (2022). A Method of Introducing Coupling Null by Shorting Pins for Stacked Microstrip Patch Antenna Array. IEEE Trans. Antennas Propag..

[69] Engheta N. (2002). An idea for thin subwavelength cavity resonators using metamaterials with negative permittivity and permeability. IEEE Antennas Wirel. Propag. Lett..

[70] Li M., Zhong B., Cheung S. (2019). Isolation enhancement for MIMO patch antennas using near-field resonators as coupling-mode transducers. IEEE Trans. Antennas Propag..

[71] He Y., Huang W., He Z., Zhang L., Gao X., Zeng Z. (2022). A Novel Cross-Band Decoupled Shared-Aperture Base Station Antenna Array Unit for 5G Mobile Communications. IEEE Open J. Antennas Propag..

[72] Lu X., Chen Y., Guo S., Yang S. (2022). An Electromagnetic-Transparent Cascade Comb Dipole Antenna for Multi-Band Shared-Aperture Base Station Antenna Array. IEEE Trans. Antennas Propag..

[73] Chang Y., Chu Q. (2021). Ferrite-Loaded Dual-Polarized Antenna for Decoupling of Multiband Multiarray Antennas. IEEE Trans. Antennas Propag..

[74] Qi X., Xu Z., Li H. (2022). High efficiency 2-D Multi-Beam Rectenna Based on Gain Enhanced Patch Array. IEEE Antennas Wirel. Propag. Lett..

[75] Wu F., Luk K. (2019). Circular Polarization and Reconfigurability of Fabry-Pérot Resonator Antenna Through Metamaterial-Loaded Cavity. IEEE Trans. Antennas Propag..

[76] Fakharian M. (2022). A high gain wideband circularly polarized rectenna with wide ranges of input power and output load. Int. J. Electron..

[77] Song C., Lu P., Shen S. (2021). Highly Efficient Omnidirectional Integrated Multiband Wireless Energy Harvesters for Compact Sensor Nodes of Internet-of-Things. IEEE Trans. Ind. Electron..

[78] Ali T., Pathan S., Biradar R. (2018). Multiband, frequency reconfigurable, and metamaterial antennas design techniques: Present and future research directions. Internet Technol. Lett..

[79] Yang P., Dang R., Li L. (2022). Dual-Linear-to-Circular Polarization Converter Based Polarization-Twisting Metasurface Antenna for Generating Dual Band Dual Circularly Polarized Radiation in Ku-Band. IEEE Trans. Antennas Propag..

[80] Maslovski S., Tretyakov S., Belov P. (2002). Wire media with negative effective permittivity: A quasi-static model. Microw. Opt. Technol. Lett..

[81] Marques R., Mesa F., Martel J., Medina F. (2003). Comparative analysis of edge- and broadside-coupled split ring resonators for metamaterial design—Theory and experiments. IEEE Trans. Antennas Propag..

[82] Caloz C., Itoh T. (2005). Electromagnetic Metamaterials: Transmission Line Theory and Microwave Applications: The Engineering Approach.

[83] Kumar P., Ali T., Pai M. (2021). Electromagnetic metamaterials: A new paradigm of antenna design. IEEE Access.

[84] Liu Z., Guo Y. (2015). Compact Low-Profile Dual Band Metamaterial Antenna for Body Centric Communications. IEEE Antennas Wirel. Propag. Lett..

[85] Li M., Luk K., Ge L., Zhang K. (2016). Miniaturization of Magnetoelectric Dipole Antenna by Using Metamaterial Loading. IEEE Trans. Antennas Propag..

[86] Abdalla M., Fouad M., Elregeily H., Mitkees A. (2012). Wideband negative permittivity metamaterial for size reduction of stopband filter in antenna applications. Prog. Electromagn. Res. C.

[87] Juan Y., Yang W., Che W. (2019). Miniaturized Low-Profile Circularly Polarized Metasurface Antenna Using Capacitive Loading. IEEE Trans. Antennas Propag..

[88] Gheethan A., Herzig P., Mumcu G. (2013). Compact 2 × 2 coupled double loop GPS antenna array loaded with broadside coupled split ring resonators. IEEE Trans. Antennas Propag..

[89] Nahar T., Rawat S. (2022). Survey of various bandwidth enhancement techniques used for 5G antennas. Int. J. Microw. Wirel. Technol..

[90] Li Y., Chen J. (2022). Design of Miniaturized High Gain Bow-Tie Antenna. IEEE Trans. Antennas Propag..

[91] Al-Bawri S., Islam M., Wong H., Jamlos M., Narbudowicz A., Jusoh M., Sabapathy T., Islam M.T. (2020). Metamaterial cell-based superstrate towards bandwidth and gain enhancement of quad-band CPW-fed antenna for wireless applications. Sensors.

[92] Zada M., Shah I., Yoo H. (2020). Metamaterial-Loaded Compact High-Gain Dual-Band Circularly Polarized Implantable Antenna System for Multiple Biomedical Applications. IEEE Trans. Antennas Propag..

[93] Bakhtiari A. (2022). Investigation of Enhanced Gain Miniaturized Patch Antenna Using Near Zero Index Metamaterial Structure Characteristics. IETE J. Res..

[94] Tang M., Ziolkowski R. (2015). Frequency-Agile, Efficient, Circularly Polarized, Near-Field Resonant Antenna: Designs and Measurements. IEEE Trans. Antennas Propag..

[95] Dey S., Dey S., Koul S. (2021). Isolation Improvement of MIMO Antenna Using Novel EBG and Hair-Pin Shaped DGS at 5G Millimeter Wave Band. IEEE Access.

[96] Selvaraju R., Jamaluddin M., Kamarudin M., Nasir J., Dahri M. (2018). Mutual coupling reduction and pattern error correction in a 5G beamforming linear array using CSRR. IEEE Access.

[97] Qamar Z., Naeem U., Khan S., Chongcheawchamnan M., Shafique M. (2016). Mutual Coupling Reduction for High-Performance Densely Packed Patch Antenna Arrays on Finite Substrate. IEEE Trans. Antennas Propag..

[98] Haykin S. (2005). Communication Systems.

[99] Mallik A., Kundu S. Design of a novel dual-band microstrip patch antenna operating at 2.45 GHz and 2.84 GHz with practical implementation. Proceedings of the 16th Int’l Conf. Computer and Information Technology.

[100] Wyndrum R. (1965). Microwave filters, impedance-matching networks, and coupling structures. Proc. IEEE.

[101] Keshavarz R., Shariati N. (2022). Highly Sensitive and Compact Quad-Band Ambient RF Energy Harvester. IEEE Trans. Ind. Electron..

[102] Eroglu A. (2022). RF/Microwave Engineering and Applications in Energy Systems.

[103] Mohan A., Mondal S. (2021). An Impedance Matching Strategy for Micro-Scale RF Energy Harvesting Systems. IEEE Trans. Circuits Syst. II Express Briefs.

[104] Wang C., Yuan B., Shi W., Mao J. (2020). Low-Profile Broadband Plasma Antenna for Naval Communications in VHF and UHF Bands. IEEE Trans. Antennas Propag..

[105] Lauder D., Sun Y. Design Considerations of Antennas and Adaptive Impedance Matching Networks for RF Energy Harvesting. Proceedings of the 2020 European Conference on Circuit Theory and Design (ECCTD).

[106] Jabbar H., Song Y., Jeong T. (2010). RF energy harvesting system and circuits for charging of mobile devices. IEEE Trans. Consum. Electron..

[107] Chong G., Ramiah H., Yin J., Rajendran J., Wong W., Mak P.I., Martins R. (2018). Ambient RF energy harvesting system: A review on integrated circuit design. Analog Integr. Circuits Signal Process..

[108] Barton T., Gordonson J., Perreault D. Transmission line resistance compression networks for microwave rectifiers. Proceedings of the IEEE MTT-S International Microwave Symposium Digest.

[109] Khan D., Oh S., Shehzad K., Basim M., Verma D., Pu Y., Lee M., Hwang K.C., Yang Y., Lee K.-Y. (2020). An Efficient Reconfigurable RF-DC Converter with Wide Input Power Range for RF Energy Harvesting. IEEE Access.

[110] Li X., Lu Y., Martins R. (2023). A 200 MHz Passive Rectifier with Active-Static Hybrid V TH Compensation Obtaining 8% PCE Improvement. IEEE Trans. Power Electron..

[111] Akram M., Ha S. (2023). A 434-MHz Bootstrap Rectifier with Dynamic *V*_TH_ Compensation for Wireless Biomedical Implants. IEEE Trans. Power Electron..

[112] Karami M., Moez K. (2019). Systematic Co-Design of Matching Networks and Rectifiers for CMOS Radio Frequency Energy Harvesters. IEEE Trans. Circuits Syst. I Regul. Pap..

[113] Lau W., Ho H., Siek L. (2020). Deep Neural Network (DNN) Optimized Design of 2.45 GHz CMOS Rectifier with 73.6% Peak Efficiency for RF Energy Harvesting. IEEE Trans. Circuits Syst. I Regul. Pap..

[114] Chong G., Ramiah H., Yin J., Rajendran J., Mak P.I., Martins R. (2021). A Wide-PCE-Dynamic-Range CMOS Cross-Coupled Differential-Drive Rectifier for Ambient RF Energy Harvesting. IEEE Trans. Circuits Syst. II Express Briefs.

[115] Almansouri A., Ouda M., Salama K. (2018). A CMOS RF-to-DC Power Converter with 86% Efficiency and −19.2-dBm Sensitivity. IEEE Trans. Microw. Theory Tech..

[116] Abouzied M., Ravichandran K., Sanchez-Sinencio E. (2017). A Fully Integrated Reconfigurable Self-Startup RF Energy-Harvesting System with Storage Capability. IEEE J. Solid-State Circuits.

[117] Cheng L., Ki W., Tsui C. (2017). A 6.78-MHz Single-Stage Wireless Power Receiver Using a 3-Mode Reconfigurable Resonant Regulating Rectifier. IEEE J. Solid-State Circuits.

[118] Zhao F., Inserra D., Gao G., Huang Y., Li J., Wen G. (2021). High-Efficiency Microwave Rectifier with Coupled Transmission Line for Low-Power Energy Harvesting and Wireless Power Transmission. IEEE Trans. Microw. Theory Tech..

[119] Shen S., Chiu C., Murch R. (2018). Multiport Pixel Rectenna for Ambient RF Energy Harvesting. IEEE Trans. Antennas Propag..

[120] Kasar Ö., Gözel M., Kahriman M. (2020). Analysis of rectifier stage number and load resistance in an RF energy harvesting circuit. Microw. Opt. Technol. Lett..

[121] Wagih M., Hilton G., Weddell A., Beeby S. (2020). Broadband millimeter-wave textile-based flexible rectenna for wearable energy harvesting. IEEE Trans. Microw. Theory Tech..

[122] Oh T., Lim T., Lee Y. (2022). A Self-Matching Rectifier Based on an Artificial Transmission Line for Enhanced Dynamic Range. IEEE Trans. Circuits Syst. I Regul. Pap..

[123] Quddious A., Zahid S., Tahir F., Antoniades M., Vryonides P., Nikolaou S. (2021). Dual-Band Compact Rectenna for UHF and ISM Wireless Power Transfer Systems. IEEE Trans. Antennas Propag..

[124] Wang Y., Zhang J., Su Y., Jiang X., Zhang C., Wang L., Cheng Q. (2022). Efficiency Enhanced Seven-Band Omnidirectional Rectenna for RF Energy Harvesting. IEEE Trans. Antennas Propag..

[125] Gozel M., Kahriman M., Kasar O. (2018). Design of an efficiency-enhanced Greinacher rectifier operating in the GSM 1800 band by using rat-race coupler for RF energy harvesting applications. Int. J. Microw.-Comput.-Aided Eng..

[126] Gyawali B., Thapa S., Barakat A., Yoshitomi K., Pokharel R. (2021). Analysis and design of diode physical limit bandwidth efficient rectification circuit for maximum flat efficiency, wide impedance, and efficiency bandwidths. Sci. Rep..

[127] Liu J., Zhang X., Xue Q. (2019). Dual-Band Transmission-Line Resistance Compression Network and Its Application to Rectifiers. IEEE Trans. Circuits Syst. I Regul. Pap..

[128] Khan W., Raad R., Tubbal F., Theoharis P., Iranmanesh S. (2024). RF Energy Harvesting Using Multidirectional Rectennas: A Review. IEEE Sens. J..

[129] Ding S., Koulouridis S., Pichon L. (2020). Implantable Wireless Transmission Rectenna System for Biomedical Wireless Applications. IEEE Access.

[130] Yang Y., Yeo J., Priya S. (2012). Harvesting Energy from the Counterbalancing (Weaving) Movement in Bicycle Riding. Sensors.

[131] Hu Y.Y., Sun S., Xu H., Sun H. (2019). Grid-Array Rectenna with Wide Angle Coverage for Effectively Harvesting RF Energy of Low Power Density. IEEE Trans. Microw. Theory Tech..

[132] Deng W., Wang S., Yang B., Zheng S. (2022). A Multibeam Ambient Electromagnetic Energy Harvester with Full Azimuthal Coverage. IEEE Internet Things J..

[133] Cai X., Geyi W., Guo Y. (2021). A Compact Rectenna with Flat-Top Angular Coverage for RF Energy Harvesting. IEEE Antennas Wirel. Propag. Lett..

[134] Lee D.J., Lee S.J., Hwang I.J., Lee W.S., Yu J.W. (2017). Hybrid Power Combining Rectenna Array for Wide Incident Angle Coverage in RF Energy Transfer. IEEE Trans. Microw. Theory Tech..

